# Acellular nerve perineurium repairs peripheral nerve injuries in a rat model via ECM-mediated barrier and optimization of the regenerative microenvironment

**DOI:** 10.1093/rb/rbag047

**Published:** 2026-03-13

**Authors:** Fulin He, Zhenpeng Li, Fawei Liao, Shuihuan Wang, Xijun Huang, Jiahui Sun, Aijun Huang, Siyu LinFang, Tao Lin, Zilong Rao, Xiaolin Liu, Shuo Tang, Qingtang Zhu, Canbin Zheng, Shuai Qiu

**Affiliations:** Department of Orthopaedics, The Seventh Affiliated Hospital of Sun Yat-Sen University, Shenzhen 518107, China; Orthopedic Center, Department of Orthopedics and Traumatology, The Affiliated Guangdong Second Provincial General Hospital of Jinan University, Guangzhou 510317, China; Department of Microsurgery, Orthopedic Trauma and Hand Surgery, The First Affiliated Hospital, Sun Yat-sen University, Guangzhou 510080, China; Department of Microsurgery, Orthopedic Trauma and Hand Surgery, The First Affiliated Hospital, Sun Yat-sen University, Guangzhou 510080, China; Department of Microsurgery, Orthopedic Trauma and Hand Surgery, The First Affiliated Hospital, Sun Yat-sen University, Guangzhou 510080, China; Medical Genetic Center, Women and Children’s Hospital, Southern University of Science and Technology, Guangzhou 510010, China; Department of Orthopedics, The Eighth Affiliated Hospital, Sun Yat-sen University, Shenzhen 518033, China; Department of Orthopedics, The Eighth Affiliated Hospital, Sun Yat-sen University, Shenzhen 518033, China; Department of Orthopedics and Traumatology, Zhujiang Hospital of Southern Medical University, Guangzhou 510280, China; PCFM Lab, GD HPPC Lab, School of Chemistry, Sun Yat-sen University, Guangzhou 510275, China; Department of Microsurgery, Orthopedic Trauma and Hand Surgery, The First Affiliated Hospital, Sun Yat-sen University, Guangzhou 510080, China; Guangdong Provincial Peripheral Nerve Tissue Engineering and Technology Research Center, Guangzhou 510080, China; Guangdong Province Engineering Laboratory for Soft Tissue Biofabrication, Guangzhou 510080, China; Department of Orthopedics, The Eighth Affiliated Hospital, Sun Yat-sen University, Shenzhen 518033, China; Biological Laboratory of Hetao Cooperation Zone, the Eighth Affiliated Hospital of Sun Yat-sen University, Shenzhen 518033, China; Department of Microsurgery, Orthopedic Trauma and Hand Surgery, The First Affiliated Hospital, Sun Yat-sen University, Guangzhou 510080, China; Guangdong Provincial Peripheral Nerve Tissue Engineering and Technology Research Center, Guangzhou 510080, China; Guangdong Province Engineering Laboratory for Soft Tissue Biofabrication, Guangzhou 510080, China; Department of Microsurgery, Orthopedic Trauma and Hand Surgery, The First Affiliated Hospital, Sun Yat-sen University, Guangzhou 510080, China; Guangdong Provincial Peripheral Nerve Tissue Engineering and Technology Research Center, Guangzhou 510080, China; Guangdong Province Engineering Laboratory for Soft Tissue Biofabrication, Guangzhou 510080, China; Department of Orthopedics, The Eighth Affiliated Hospital, Sun Yat-sen University, Shenzhen 518033, China; Biological Laboratory of Hetao Cooperation Zone, the Eighth Affiliated Hospital of Sun Yat-sen University, Shenzhen 518033, China

**Keywords:** peripheral nerve injury, nerve regeneration, regenerative microenvironment, acellular nerve perineurium, extracellular matrix

## Abstract

Building upon the clinically established platform of acellular nerve allografts (ANAs), we developed an advanced derivative: acellular nerve perineurium (ANP) grafts. These grafts are characterized by their preservation of the native perineurial barrier architecture and a unique extracellular matrix (ECM) composition, distinct from the endoneurial basement membrane. This distinctive ECM profile endows ANPs with significantly enhanced barrier integrity and robust neuroprotective properties. *In vitro* evaluations confirmed that ANP provides a highly favorable substrate, effectively supporting the adhesion and active proliferation of dorsal root ganglion neurons. In a rat model of sciatic nerve transection, ANP grafts demonstrated remarkable therapeutic efficacy. They markedly inhibited the deposition of chondroitin sulfate proteoglycans (CSPGs) at the repair site, thereby preventing traumatic neuroma formation. Furthermore, ANP treatment resulted in a doubling of regenerated axon density and a significant increase in target muscle action potential amplitude. Behavioral recovery in ANP-treated animals approached the functional levels observed in crush-injury controls. Multiomics analyses provided mechanistic insight, revealing that ANP-mediated repair activates multiple pro-regenerative signaling pathways. These collective findings position ANP grafts as a highly promising and clinically translatable biomaterial strategy for improving functional outcomes in peripheral nerve repair.

## Graphical Abstract

**Graphical Abstract:**
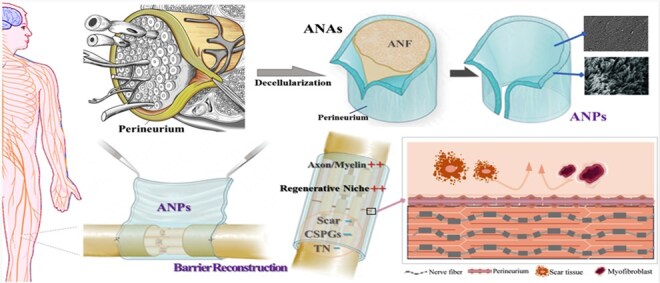

## Introduction

Traumatic peripheral nerve injury (TPNI), caused by mechanical trauma, is a major cause of limb disability worldwide and remains a formidable challenge in reconstructive surgery. Pathophysiological barriers—including disordered axonal regeneration, scar hyperplasia, traumatic neuroma (TN) formation, microstructural disruption and a persistently hostile regenerative microenvironment—limit functional recovery [1–3]. The socioeconomic impact is substantial; in the United States alone, treatment of upper and lower limb TPNIs costs more than $9.7 billion annually [4]. Because of the limited intrinsic regenerative capacity of the peripheral nervous system (PNS) and prolonged treatment courses, many patients experience unsatisfactory outcomes, which place heavy burdens on both patients and surgeons [5]. These challenges highlight the need for therapeutic strategies that not only enhance axonal regeneration but also reconstruct native nerve architecture and optimize the post-traumatic microenvironment.

To address these issues, our team at Sun Yat-sen University developed acellular nerve allografts (ANAs), which have been widely adopted in clinical practice in China [6, 7]. Clinical studies confirm that ANAs are reliable grafts for repairing peripheral nerve defects up to 7 cm in length [8]. Their efficacy is derived from two features: (i) preservation of the hierarchical microstructure of peripheral nerves, providing microstructural biomimicry; and (ii) retention of bioactive ECM components that promote axonal growth, providing microenvironmental biomimicry. Following the clinical translation of ANAs, we conducted ‘reverse research’ to elucidate the mechanisms of ANA-mediated repair. These efforts included defining the roles of specific ECM proteins [9], characterizing regional microarchitecture within ANAs [10] and exploring ANA use in suppressing TN formation at nerve stumps [11]. We found that ANAs preserve both the ECM framework of nerve fascicles (endoneurial-basement membrane complexes) and the barrier function of the perineurium. Implanted fascicles guide axonal extension via ECM-driven remodeling, whereas the perineurium shields regenerating axons from infiltration by myofibroblasts, scar tissue and aberrant vasculature. This barrier effect reduces α-smooth muscle actin (α-SMA) expression and disrupts the pathological microenvironment that drives TN formation [11]. TNs arise from maladaptive interactions between injured nerve stumps and surrounding tissue, often leading to poor target organ reinnervation and neuropathic pain. Local disruption of the ECM microenvironment can lead to alterations in tissue structure, stiffness and immune responses, which may hinder the healing of pathological conditions [12, 13]. Unlike the epineurium, the perineurium forms the peripheral nerve-blood barrier, maintaining intraneural homeostasis and protecting nerves from hematoma and fibrotic invasion [14, 15]. Clinically, perineurial integrity is correlated with improved outcomes in TPNI patients. These findings suggest that isolated acellular nerve perineurium (ANP) grafts derived from ANAs may provide substantial microenvironmental protection during nerve regeneration.

Despite these insights, no study has systematically examined the distinct biological functions of different anatomical regions of ANAs, and the properties of ANPs remain largely unknown. Preliminary work from our group demonstrates that decellularization preserves the boundaries between fascicles, perineurium and epineurium, making complete perineurial isolation technically feasible [11, 16]. Accordingly, this study investigates the composition, microstructure and regenerative niche-modulating functions of ANPs to evaluate their potential as a surgical biomaterial for enhancing both axonal regeneration and functional recovery following TPNI.

## Materials and methods

### Animals

All animal procedures were approved by the Animal Ethics Committee of Sun Yat-sen University (Approval No. SYSU-IACUC-2024-000872; 10 March 2024) and conducted in accordance with the ARRIVE guidelines. Efforts were made to minimize animal suffering, and all procedures complied with the National Research Council’s Guide for the Care and Use of Laboratory Animals. Animals had free access to standard rat chow and water throughout the study.

### Preparation and characterization of ANPs

ANPs were derived from the sciatic nerves of adult beagle dogs humanely euthanized after surgical training. Bilateral sciatic nerves were excised and subjected to a decellularization protocol to produce decellularized nerve scaffolds. Briefly, nerves were immersed overnight in distilled water, followed by sequential chemical extraction: agitation in 4% (w/v) Triton X-100 (Sigma-Aldrich) for 12 h and 4% (w/v) sodium deoxycholate (Sigma-Aldrich) for an additional 12 h. After thorough rinsing with phosphate-buffered saline (PBS), the grafts were sterilized by Co^60^ irradiation and sectioned into 5-mm segments for subsequent experiments.

First, three segments of ANAs were cryosectioned into 4-μm-thick slices using a cryostat (CM 1950, Leica, Germany) for *ex vivo* DRG culture experiments. Another five ANA segments were reserved for subcutaneous implantation in SD rats. Second, the remaining ANA segments were further dissected into ANFs and ANPs via optical microscopy. To evaluate the physicochemical properties of ANPs, standard hematoxylin and eosin (H&E) staining and Masson’s trichrome staining were performed on ANP sections. Stained images were acquired using a Pannoramic SCAN slide scanner (3DHISTECH, Hungary), and the microstructures of the ANPs were examined by scanning electron microscopy (SEM; Regulus8100, Hitachi, Japan). Third, six segments each of ANFs and ANPs were cryopreserved at −80°C for subsequent proteomic analysis. Finally, at least 15 ANP segments were collected for sciatic nerve repair experiments in SD rats following nerve injury.

Mechanical property determination: The mechanical properties of ANAs and ANPs were tested using a universal testing machine (China, ZQ-990B). The samples used for tensile testing were wet cylindrical specimens (with a diameter of 3 mm and a height of 15 mm), and the tensile speed was fixed at 20 mm/min. All tests were conducted in a constant-temperature environment at 37°C. Tensile strength and elongation at break: Cylindrical ANA or ANP samples (*n* = 3) were directly clamped at both ends without suturing and subjected to uniaxial tensile testing until fracture. Suture pull-out strength: Before testing, a single 8-0 Prolene suture was used to suture and secure the epineurium at one end of the ANAs and one end of the ANPs, respectively, following the same surgical suturing method. Subsequently, the suture loop was fixed to the upper clamp of the testing machine, while the main body of the sample was secured to the lower clamp for vertical pull-out testing.

### Proteomics analysis

In this study, we conducted proteomic analyses of two sets of tissue samples from different sources. The first set included ANF and ANP tissues meticulously isolated from ANAs via optical microscopy. The proteomic data analysis for this group was performed by Shanghai Lu Ming Biotechnology Co., Ltd. (Shanghai, China). The second set comprised postoperative anastomotic nerve tissues from the Con (control) and ANP groups in a sciatic nerve injury repair experiment using SD rats, with proteomic data analysis conducted by Guangzhou Gidio Biotechnology Co., Ltd. All the tissues used for proteomic analysis were stored and transported in preservation solution at −80°C.

The sample processing workflow was as follows: After protein digestion, quantification and quality control steps, samples from each group were labeled with TMT reagents (Thermo Fisher Scientific, USA). Liquid chromatography–tandem mass spectrometry (LC-MS/MS) analysis was subsequently performed for 60 or 90 min using a Q Exactive mass spectrometer (Thermo Fisher Scientific) coupled with an easy nLC system (Thermo Fisher Scientific). All the raw data were systematically searched using Proteome Discoverer software (version 2.4), with the UniProt Mus musculus database used as the reference. During database searching, trypsin-specific digestion conditions were applied and cysteine alkylation was set as a fixed modification. Protein quantification was performed using the TMT/iTRAQ technique. The global false discovery rate (FDR) was strictly controlled at 0.01, and only proteins containing at least one unique peptide were included in the quantitative analysis. Hierarchical clustering analysis was conducted using Cluster 3.0 and Java Treeview software. Additionally, InterProScan software in combination with the Pfam database was used to search protein sequences for domain characterization. The reliability of peptide identification was validated using the PeptideProphet algorithm (FDR < 1%), and DEPs were screened on the basis of the criteria of FC > 2 and *P* < 0.05. All identified proteins were functionally annotated using the Gene Ontology (GO) database (http://www.blast2go.com/b2ghome; http://geneontology.org/) and the Kyoto Encyclopedia of Genes and Genomes (KEGG) pathway database (http://www.genome.jp/kegg/). Further GO and KEGG enrichment analyses were performed on the DEPs. Additionally, the MatriSomeDB database (https://matrixdb.univ-lyon1.fr/) was utilized to screen and analyze ECM proteins and their interactions.

### *Ex vivo* DRG culture and immunofluorescence staining

Following the preparation of ANAs, transverse sections of 4 μm thickness were obtained for *ex vivo* DRG culture experiments. Briefly, gelatin sponge (B-type, 3 × 3 × 2 mm; FastCare brand; Jiangxi Zhongqiang Industrial Co., Ltd., China) and ANAs slices were immobilized at the bottom of culture dishes with Matrigel (Corning, NY), a thermosensitive neural culture gel matrix. DRGs were subsequently dissected from postnatal day 1 (P1) Sprague–Dawley rats and collected in cold DMEM/F12. Under a stereomicroscope, the excess roots of the DRGs were carefully removed, and ten intact DRGs were seeded onto the ANP region of the ANAs. The DRGs were then incubated in neurobasal medium (Gibco, Grand Island, NY, USA) supplemented with 2% B27, 0.3% L-glutamine (Gibco, Grand Island, NY, USA), and 100 ng/mL nerve growth factor (NGF; Peprotech, Rocky Hill, NJ, USA) at 37°C in a humidified incubator with 5% CO_2_. The medium was replaced every other day, and all procedures were performed under sterile conditions. After a 48-h incubation period, the medium and coverslips were removed, and the adhered ANAs and DRGs at the bottom of the culture dishes were subjected to immunofluorescence staining to assess neurite outgrowth. Specifically, DRG explants were fixed in 4% paraformaldehyde for 20 min, washed three times with PBS and blocked with QuickBlock solution (Beyotime) at room temperature for 30 min. The samples were subsequently incubated overnight at 4°C with primary antibodies against neurofilament 200 (1:200, Sigma–Aldrich) and S100B (1:200, Sigma–Aldrich). After three washes with PBS, secondary antibodies—including Alexa Fluor 488-conjugated goat anti-rabbit IgG (1:1000, Invitrogen) and Alexa Fluor 647-conjugated goat anti-mouse IgG (1:1000, Invitrogen)—were added, and the samples were incubated at room temperature for 1 hour. Nuclear staining was performed with DAPI (Thermo Fisher) for 15 min at room temperature. Finally, the DRG explants were imaged using a Zeiss LSM 900 laser scanning confocal microscope (LSM980 Airyscan2, Zeiss, Germany). To verify the reliability and reproducibility of the experimental results, five independent replicate experiments (*n* = 5) were conducted under identical conditions for the culture and staining procedures.

### Animal grouping and surgical procedures

Specific pathogen-free (SPF)-grade female SD rats (strain: Sprague–Dawley, age: 8 weeks, weight: 200–250 g; obtained from the Laboratory Animal Center of Sun Yat-sen University, Guangzhou, China) were used in this study. The experiments involving SD rats were divided into two parts. In the first part, five SD rats were used to evaluate tissue infiltration following the implantation of ANAs. Briefly, after anesthesia was induced via intraperitoneal injection of sodium pentobarbital (50 mg/kg), the dorsal hair was shaved and ANAs were subcutaneously implanted into the dorsal region under aseptic conditions. The skin incision was closed using 4–0 sutures. In the second part, 27 SD rats were randomly divided into three groups: the clamp injury (CI) group (*n* = 5), the control (Con) group (*n* = 11) and the ANP group (*n* = 11). In each group, nerve samples from 5 rats were collected for histological staining, electrophysiological assessment and behavioral evaluation. Additionally, nerve samples from the remaining six rats in the Con and ANP groups were subjected to proteomic analysis. Anesthesia was similarly induced via intraperitoneal injection of sodium pentobarbital (50 mg/kg). Under aseptic conditions, the right sciatic nerve was exposed. The origin of the posterior gluteal nerve branch at the sciatic notch level was identified to determine the site of nerve injury. In the CI group, all the right sciatic nerves were clamped for 10 s at a consistent pressure using the first occlusal joint of the same forceps. In the other two groups, the sciatic nerve was sharply transected and meticulously sutured under a surgical microscope (Leica, Wetzlar, Germany) using 10–0 sutures. In the ANP group, the anastomotic site of the sutured sciatic nerve was enveloped with ANAs, whereas no additional treatment was applied to the Con group. In all the groups, the muscle layer was left intact and the skin incision was closed with 4–0 sutures.

### Assessment of neural regeneration function

Functional recovery was evaluated at 2-, 4- and 6-weeks post-surgery using the sciatic function index (SFI). Specifically, the rats were sequentially placed in a transparent acrylic chamber (50 × 50 × 50 cm) with a video camera positioned below to record their gait for at least 30 s per rat. Only the data from uninterrupted gait cycles (from heel strike to subsequent heel strike of the same limb) during steady, straight-line walking were included in the analysis. The paw length (PL), toe spread (TS) and intermediary toe spread (IT) of both the normal (N) and experimental (E) hindlimbs were measured. The SFI was calculated using the following formula: SFI = −38.3 × [(EPL − NPL)/NPL] + 109.5 × [(ETS − NTS)/NTS] + 13.3 × [(EIT − NIT)/NIT] − 8.8.

### Electrophysiological evaluation and histological examination of the gastrocnemius muscle

Following functional assessment, five SD rats from each group were anesthetized with sodium pentobarbital (50 mg/kg). The right sciatic nerve was re-exposed via the original incision. A bipolar stimulating electrode was placed proximally to deliver a single electrical stimulus, while a recording electrode was inserted into the belly of the gastrocnemius muscle. Electrical stimulation (15 V amplitude, 0.01 ms pulse duration) was applied to the proximal nerve trunk, and compound muscle action potentials (CMAPs) and CMAP latency were recorded ipsilaterally using a biofunctional experimental system (BL-420F, Techman, Chengdu, China). This procedure was repeated three times. The gastrocnemius muscle and the repaired sciatic nerve from each group were subsequently harvested, and the total wet weight of the gastrocnemius muscle was subsequently measured. Paraffin-embedded sections of the mid-belly region of the gastrocnemius muscle were prepared and subjected to Masson’s trichrome staining, followed by light microscopy examination. Images of ten randomly selected fields per sample were captured using an Axio Scan Z1 slide scanner (Goettingen, Germany) and analyzed with ImageJ software (NIH, Bethesda, Maryland, USA) to quantify the cross-sectional areas of collagen and muscle fibers. The percentage of collagen fibers was calculated as the ratio of the collagen fiber area to the sum of the collagen and muscle fiber areas.

### Specimen preparation and histological evaluation

Following electrophysiological assessment, the right sciatic nerves from five SD rats per group were harvested for histological evaluation. Longitudinal sections of approximately 4 mm around the anastomosis site were prepared and subjected to Masson’s trichrome staining, Sirius red staining, silver staining and immunohistochemistry for α-SMA, claudin-1 and Glut-1, as well as immunofluorescence for neurofilament-200 (NF-200) and CSPG expression. Additionally, specimens from the distal tibial and common peroneal nerves were separately collected (1 μm), stained with toluidine blue, and examined by transmission electron microscopy (TEM). Histological and TEM samples were preserved in 4% paraformaldehyde. After fixation, the 4-mm segments surrounding the anastomosis were processed using standard paraffin-embedding techniques and sectioned into 4-μm slices. To ensure standardized staining regions, sections were randomly selected from depths of 1000–1200 μm beneath the sample surface. Following the exclusion of small or poorly cut sections, at least 30 slices were obtained from each sample. Thirty sections were then randomly allocated, with every fifth section designated for Masson’s trichrome staining, Sirius red staining, silver staining and immunohistochemistry for α-SMA, Claudin-1 and Glut-1.

For immunohistochemical staining of α-SMA, Claudin-1 and Glut-1, tissue sections were incubated for 2 hours at 37°C with the following primary antibodies: mouse anti-α-SMA (1:1000 dilution, Cyagen Biosciences, Cat# GB111364), rabbit anti-Claudin-1 (1:800 dilution, Abcam, Cat# ab115783) and anti-Glut-1 (1:200 dilution, Arigo Biolaboratories, Cat# ARG10781). The sections were blocked with goat serum (Solarbio) for 10 min, rinsed with 0.01 M phosphate-buffered saline (PBS, pH 7.4), and incubated for 1 h at 26°C with biotinylated secondary antibodies: goat anti-mouse IgG (1:200 dilution, Servicebio, Cat# GB23301) and goat anti-rabbit IgG (1:200 dilution, Servicebio, Cat# GB25303). Immunoreactivity was detected by incubating the sections with horseradish peroxidase (HRP)-conjugated streptavidin (1:200 dilution, Servicebio, Cat# G3431) and 3,3′-diaminobenzidine (DAB; Beyotime, Shanghai, China). All the tissue sections were quantitatively analyzed using ImageJ software (v1.50i; National Institutes of Health, Bethesda, MD, USA). For immunofluorescence analysis of NF-200 and CSPG expression, tissue sections were rehydrated with PBS and blocked for nonspecific binding using QuickBlock solution (Beyotime). The sections were incubated overnight at 4°C with the following primary antibodies: mouse anti-rat neurofilament-H (NF-200, 1:200 dilution, BioLegend) and rabbit anti-rat NG2 (CSPG4, 1:500 dilution, Abcam). After three washes with PBS, the sections were incubated for 1 hour at room temperature with the following secondary antibodies: Alexa Fluor 647-conjugated goat anti-rabbit IgG (1:1000 dilution, Invitrogen) and Alexa Fluor 488-conjugated goat anti-mouse IgG (1:1000 dilution, Invitrogen). Finally, the sections were counterstained with DAPI (Thermo Fisher Scientific) for 15 min at room temperature. Images were acquired using a Leica DMi8 inverted fluorescence microscope and quantitatively analyzed with ImageJ software (v1.50i; NIH, Bethesda, MD, USA).

### Toluidine blue staining and transmission electron microscopy

To evaluate the effects of ANP repair on axonal extension in the sciatic nerve stump and ECM organization, toluidine blue staining and TEM were employed to examine the ultrastructure of regenerated distal tibial and common peroneal nerve samples. Specifically, 2-mm distal nerve segments were immediately fixed in 2.5% glutaraldehyde for 3 h after extraction, followed by postfixation in 1% osmium tetroxide for 2 h. The samples were subsequently dehydrated through a graded ethanol series, embedded in epoxy resin and sectioned into 1-μm semithin and 50-nm ultrathin slices. The semithin sections were stained with toluidine blue, imaged using an Axio Scan Z1 slide scanner (Carl Zeiss, Göttingen, Germany) and analyzed with Zen 2.3 Blue Edition software (Carl Zeiss Microscopy). The ultrathin sections were stained with 3% uranyl acetate and lead citrate, and images were captured using transmission electron microscopy (TEM, JEOL, Tokyo, Japan).

### Statistical analysis

Image editing and processing were performed using Photoshop software (Adobe Systems, version 2019). Image data acquisition was conducted with ImageJ software (National Institutes of Health, USA). Quantitative data were presented as the mean ± standard deviation (SD) and were analyzed with SPSS 27.0 statistical software (SPSS Inc., Chicago, IL, USA). For continuous variables conforming to a normal distribution and homogeneity of variance, intergroup comparisons were performed using the unpaired Student’s *t* test, whereas multigroup comparisons were conducted with one-way analysis of variance (ANOVA) followed by Bonferroni *post hoc* correction. Statistical significance was considered if *P *< 0.05.

## Results

### Characterization of ANPs

**Figure 1:**
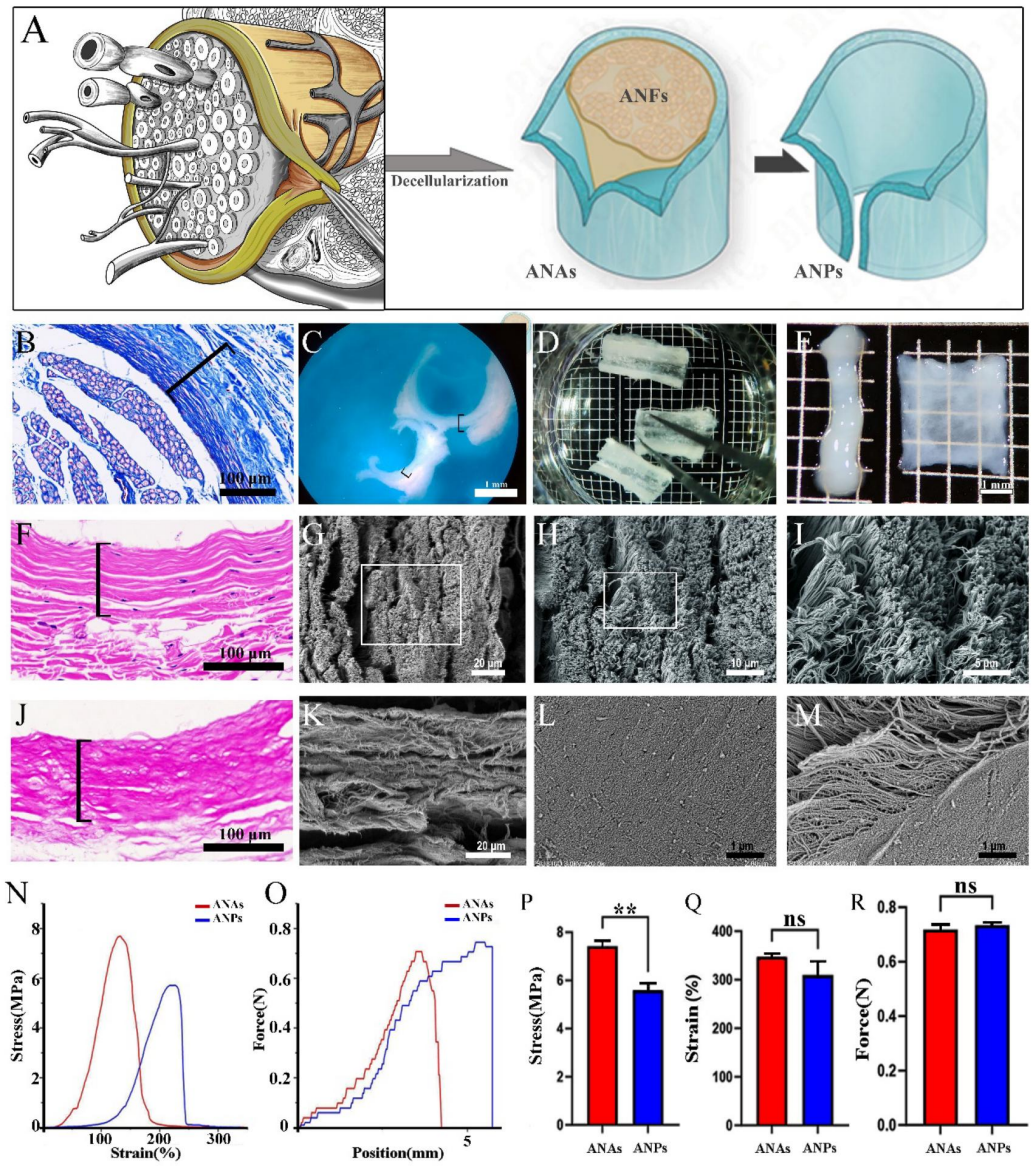
Morphological—microstructural features and mechanical performance of ANPs. (**A**) Schematic representation of peripheral nerve anatomy, showing myelinated axons bundled and surrounded by the perineurium (highlighted in yellow and grasped by forceps). After decellularization, the ANAs scaffold can be obtained and through microdissection, the ANPs can be acquired. (**B**) Masson’s trichrome staining of a transverse section of fresh nerve. (**C**) Cross-sectional appearance of hydrated ANPs (brackets); the external epineurium remains partially attached and is removed in subsequent processing. (**D**) Longitudinal view of opened ANPs. (**E**) Gross comparison of hydrated acellular nerve fascicle (ANF, left) and unfolded hydrated ANPs (right). (**F**) H&E staining of a transverse section of fresh nerve, with the perineurium layer indicated by brackets. (**G**–**I**) SEM images of ANPs in transverse section, showing densely aligned longitudinal ECM fibers at progressively higher magnification. (**J**) H&E staining of a transverse section of ANPs, with brackets marking the perineurium layer. (**K**) SEM image of a sagittal section of ANPs. (**L**, **M**) SEM images of the inner ANP surface, illustrating smooth (L) and less smooth (M) ECM regions. (**N**, **O**) Tensile strength (N) and suture pull-out strength(O) testing of ANAs and ANPs. (**P**–**R**) Quantitative analysis of tensile strength(P), elongation at break(Q) and suture pull-out strength(R) of ANAs and ANPs. (*n* = 3 per group; unpaired Student’s *t*-test; data were presented as mean ± SD; *P < 0.05, **P< 0.01, ***P < 0.001; ns: not significant). Scale bars: B, F, J = 100 µm; C, E = 1 mm; G, K = 20 µm; H = 10 µm; I = 5 µm; L, M = 1 µm.

To determine the potential of ANPs in improving the outcomes of peripheral nerve repair, we first aimed to characterize the microstructure and ECM composition of ANPs using morphological and histological analyses. The experiments were conducted using segments of sciatic nerve ANAs from beagle dogs, from the point where the main trunk bifurcates exclusively into the tibial nerve and common peroneal nerve. The perineurium, forming the elastic outer layer of each nerve fascicle, was identified by microscopy (Figure 1A). Masson staining of fresh nerve cross-sections revealed that the perineurium, which surrounded densely packed myelinated fibers, had a thickness of approximately 100 μm (Figure 1B). Through chemical extraction and microsurgical dissection, ANPs were obtained, and only residual fragmented decellularized epineurium remained on the outer surface. The ANPs exhibited a dense and resilient texture, appearing semitranslucent (Figure 1C). After removal of the residual fragmented epineurium and subsequent lyophilization, tubular ANPs were obtained (Figure 1D), which were fully flattened into sheet-like structures (Figure 1E). H&E staining of fresh nerve cross-sections revealed abundant nuclei of epithelial cells within the perineurium (Figure 1F). SEM imaging of ANP cross-sections revealed densely aligned longitudinal ECM fibers (Figure 1G–I). H&E staining of ANP cross-sections confirmed the complete removal of all the cellular nuclei from the perineurium (Figure 1J). SEM imaging of longitudinal ANP sections revealed a multilayered, tightly assembled ECM structure (Figure 1K). The inner surface of the ANPs appeared smooth and compact (Figure 1L), whereas in some regions, ruptured inner walls exposed abundant nanoscale ECM fibers (Figure 1M). These structural features indicate that ANPs preserve the native perineurial barrier architecture while providing a robust, cell-free scaffold suitable for further investigation in peripheral nerve repair. The tensile strength, elongation at break and suture retention strength of the fabricated ANAs and ANPs were quantitatively assessed (*n* = 3 per group; Figure 1N–R). In terms of tensile strength, the tensile strength of ANP is 5.58 ± 0.30 MPa, which is significantly lower than that of ANAs scaffolds (7.42 ± 0.23 MPa), showing a decrease of approximately 24.9% (*P *< 0.05). This result suggests that ANP is a core structural component in maintaining the overall mechanical strength of ANAs. In terms of ductility, both ANP (elongation at break 309.4 ± 28.4%) and ANAs (347.0 ± 7.2%) exhibit excellent deformation capabilities, with no statistically significant difference between them. This indicates that ANP possesses elasticity compatible with neural tissue, allowing it to provide effective buffering by substantial elongation when nerves are stretched, thereby protecting damaged nerves from mechanical injury. For the critical surgical indicator—suture retention strength—ANP (0.734 ± 0.011 N) and ANAs (0.734 ± 0.011 N) perform consistently, with no statistically significant difference. This demonstrates that when using ANP for suturing in nerve surgery, its mechanical reliability is comparable to that of ANAs, and surgeons need not worry about suture failure due to insufficient intrinsic mechanical strength.

### TMT-labeled proteomic analysis of ANPs and ANFs

**Figure 2:**
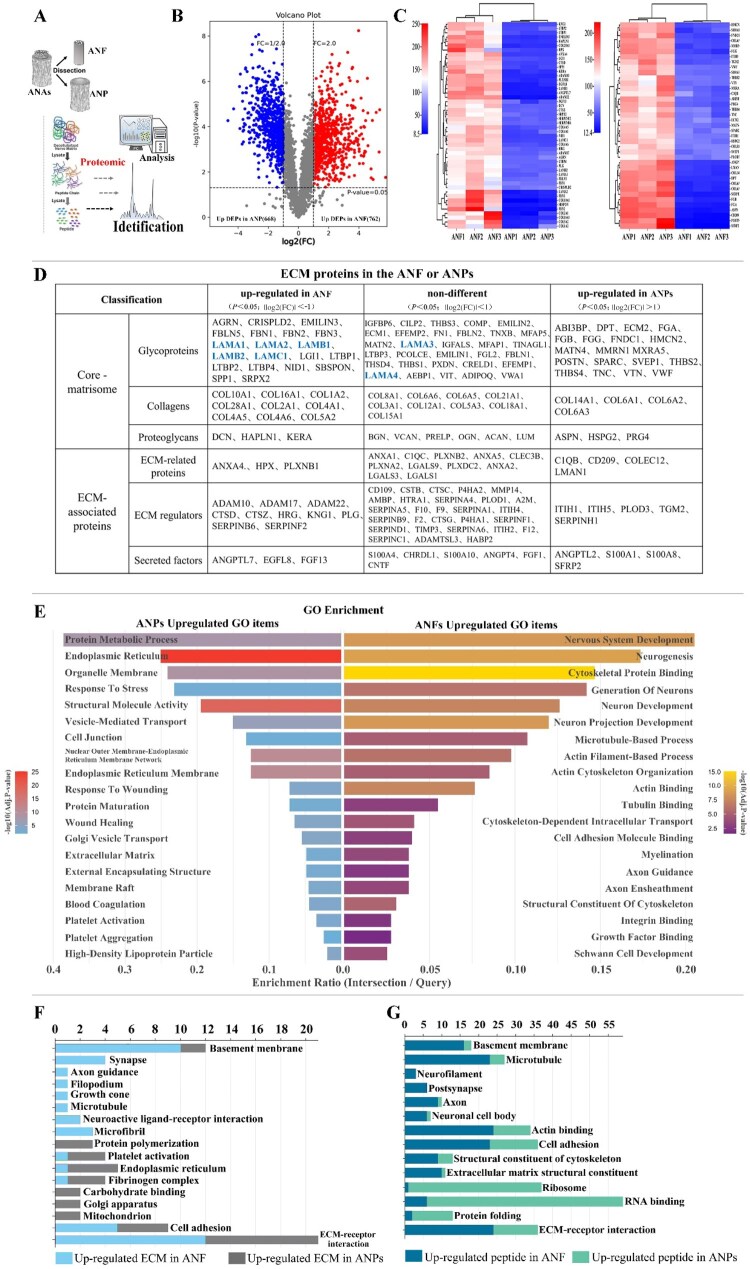
Proteomic and bioinformatics profiling of ANAs. (**A**)Schematic showing the separation of an ANA into an ANF core and an ANP layer for proteomic and bioinformatics analysis. (**B**) Volcano plot showing all the expressed proteins in two groups, the different expressed proteins (DEPs) were defined as *P*-value < 0.05 and |log2(FC)| > 1. The red dots represent 762 upregulated DEPs in the ANF samples and the blue dots represent 668 upregulated DEPs in the ANP samples. (**C**) Heatmap of ANF vs. ANP proteomes: ECM proteins with higher abundance in ANF samples are shown in the left panel and those enriched in ANP samples in the right panel. (**D**) Classification and detailed ECM composition of ANF and ANP samples. (**E**) Results of Gene Ontology (GO) analysis about the DEPs in two groups. The left part of the graph shows the significantly enhanced GO items in ANP samples, while the right part of the graph shows the significantly enhanced GO items in ANF samples. (**F**, **G**) Functional Characteristics. GO functional enrichment and KEGG pathway analysis comparing ECM components and total peptide profiles, respectively, between ANF and ANP. Pillar length corresponds to the number of differentially expressed proteins (DEPs) associated with each functional category.

Identifying and quantifying ECM components, along with analyzing ECM protein network interactions, are critical steps in understanding the role of ECM in tissue engineering. Here, we employed multiplexed TMT 10-plex proteomics analysis to generate quantitative proteomic profiles for five categories of ECM components, elucidating compositional differences between ANF and ANP tissues in ANAs. ANA tissues were microsurgically dissected to obtain ANP (*n* = 3) and ANF (*n* = 3) samples (Figure 2A). A total of 4386 protein peptides were identified with a false discovery rate (FDR) <1%, among which 1430 showed significant differential expression: 762 upregulated in ANFs and 668 upregulated in ANPs (Figure 2B). Of the 1028 ECM-related proteins detected, 87 were significantly differentially expressed: 48 upregulated in ANFs, and 39 upregulated in ANPs (Figure 2C). To delineate functional disparities between ANP-ECM and ANF-ECM, we performed a comparative analysis of ECM types and abundances using MatriSomeDB, with key laminin peptides of the basement membrane highlighted in blue (Figure 2D). The basement membrane-related components in ANF tissue, such as LAMA1, LAMA2, LAMB1, LAMB2 and LAMC1, are present in higher quantities compared to those in ANP tissue. The results of GO enrichment analysis, highlighting the biological processes and molecular functions significantly upregulated in ANP and ANF samples, respectively. (Figure 2E) Left Column (ANPs Upregulated GO Items): Terms are predominantly associated with protein metabolism and synthesis, including ‘Protein Metabolic Process’, ‘Endoplasmic Reticulum’, ‘Protein Maturation’ and ‘Vesicle-Mediated Transport’. Additional enriched terms relate to cellular stress and repair responses, such as ‘Response to Wounding’ and ‘Wound Healing’, as well as extracellular structure and membrane domains, exemplified by ‘Extracellular Matrix’, ‘External Encapsulating Structure’ and ‘Membrane Raft’. Terms like ‘Blood Coagulation’, ‘Platelet Activation’ and ‘Platelet Aggregation’ further suggest involvement in coagulation and inflammatory processes. Right Column (ANFs Upregulated GO Items): Significant enrichment is observed in terms closely linked to nervous system development and function, such as ‘Neurogenesis’, ‘Generation of Neurons’, ‘Neuron Development’ and ‘Neuron Projection Development’. There is also strong representation of cytoskeletal organization and binding activities, including ‘Cytoskeletal Protein Binding’, ‘Actin Binding’, ‘Tubulin Binding’ and processes like ‘Actin Cytoskeleton Organization’ and ‘Microtubule-Based Process’. Furthermore, terms such as ‘Axon Guidance’, ‘Axon Ensheathment’, ‘Myelination’ and ‘Schwann Cell Development’ indicate a specific role in axon formation and glial support. Functional terms like ‘Integrin Binding’ and ‘Growth Factor Binding’ suggest enhanced potential for cell adhesion and signaling. GO functional and KEGG pathway analyses revealed distinct functional characteristics for differentially expressed ECM components between ANPs and ANFs (Figure 2F and G). For streamlined interpretation, we focused on neurodevelopment-associated pathways, categorized as follows: Class I: directly related to neural development (basement membrane, synapses, axon guidance, filopodia, growth cones, microtubules, neuroactive ligand–receptor interactions, microfibrils) and Class II: involved in biosynthesis (protein polymerization, platelet activation, endoplasmic reticulum, fibrinogen complex, carbohydrate binding, Golgi apparatus, mitochondria, cell adhesion and ECM–receptor interactions). The results demonstrated that the ECM proteins upregulated in ANPs (e.g. ECM, VTN, VWF and ITIH) were predominantly associated with Class II pathways, whereas those upregulated in ANFs (e.g. LAM, FBN, DCN and ADAM) were enriched in Class I pathways (Figure 2F). A similar trend was observed for whole-proteome peptides: ANP-upregulated peptides clustered in Class II, whereas ANF-upregulated peptides aligned with Class I (Figure 2G). TMT-based proteomic profiling revealed that ANPs are enriched in biosynthesis- and matrix-support-related ECM proteins, whereas ANFs predominantly contain neurodevelopment-associated ECM components, indicating distinct functional specializations within ANA-derived tissues.

### ANP scaffolds support DRGs growth *in vitro*

**Figure 3:**
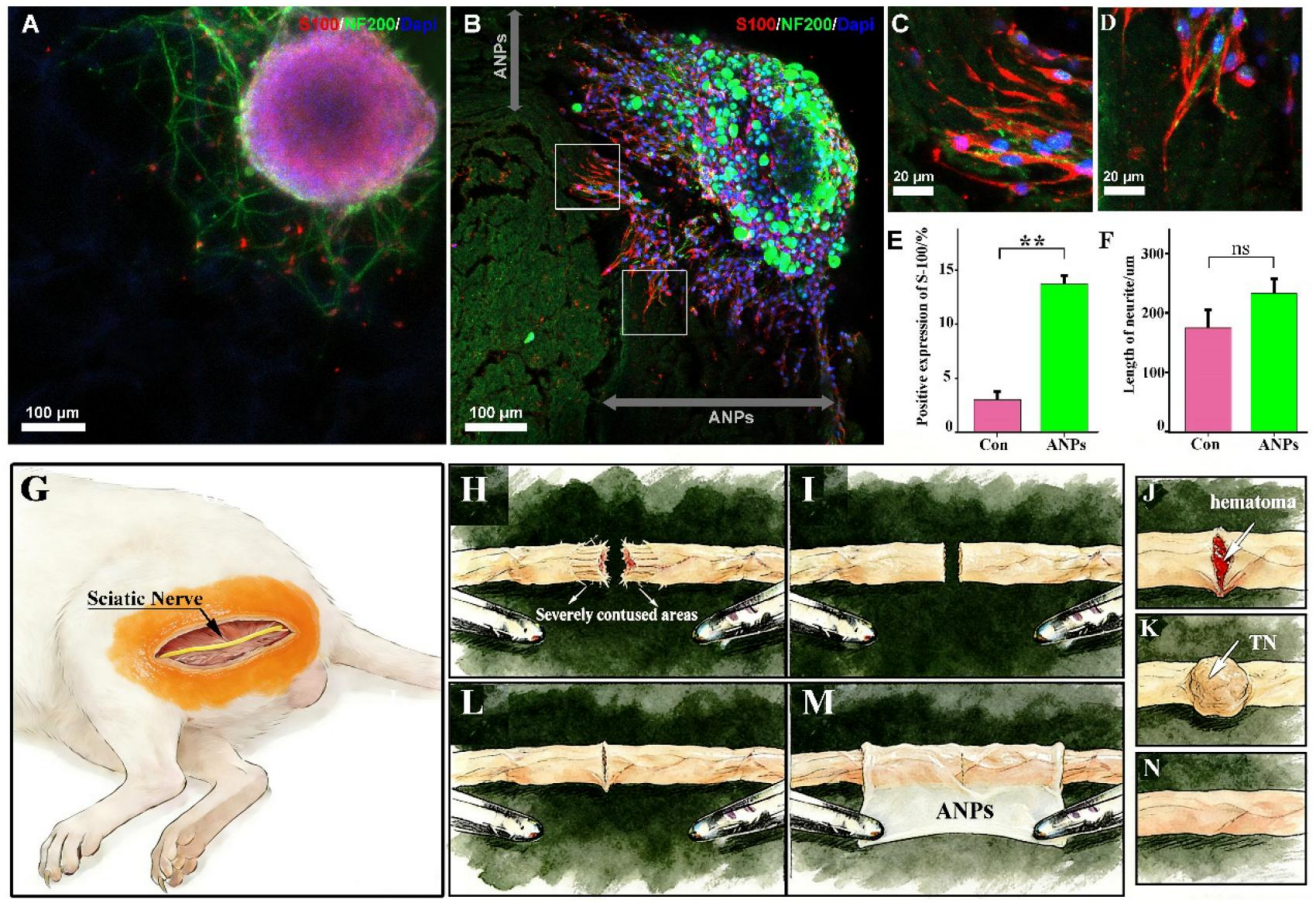
*In vivo* and *in vitro* experimental studies on ANPs. (**A**) DRGs adhered to the gelatin matrix scaffold. (**B**) DRGs adhered to the ANP region of the scaffold and extended aligned neurites along its matrix (gray double-headed arrow indicates the ANP region), indicating a supportive microenvironment for neural outgrowth. (**C**, **D**) Higher magnification views of boxed areas in (A), revealing Schwann cell migration and neurite infiltration into the ANP scaffold, further demonstrating scaffold biocompatibility and guidance potential. (**E**, **F**) Quantitative analysis of the percentage of S100-positive fluorescent area and the length of neurite in control versus ANP groups. (*n* = 5 per group; unpaired Student’s *t*-test; data were presented as mean ± SD; *P < 0.05, **P < 0.01, ***P < 0.001, ns: not significant). (**G**) Schematic diagram of the SD rat sciatic nerve main trunk injury repair model. (**H**) Schematic diagram showing severe local contusion after nerve transection and the need for appropriate local trimming. (**I**) Schematic diagram showing the neat nerve stumps obtained after appropriate local trimming following nerve transection. (**J**, **K**) Schematic diagram showing local hematoma accumulation in the early stage and TN formation in the late stage after nerve suturing. (**L**, **M**) Schematic diagram showing neat anastomosis after nerve transection and covering the injury site with ANP. (**N**) Schematic diagram illustrating the achievement of scarless repair after nerve suturing. Scale bars: A = 100 µm; B, C = 20 µm. Scale bars: D−F, I−K = 1 mm.

To comprehensively evaluate the neuroregenerative potential of ANP scaffolds, dorsal root ganglia (DRGs) were cultured on the gelatin matrix scaffold (control group) and ANP region of ANA-derived tissues for 48 h. In the control group, immunofluorescence staining revealed limited neurite outgrowth (NF200, neuronal marker), along with fewer S100-labeled Schwann cells exhibiting restricted morphological extension (Figure 3A). The three-dimensional fibrous architecture of the ANP scaffold provided an organized substrate for both neuronal and glial elements. Immunofluorescence staining for neurofilament protein revealed extensive neurite outgrowth radiating from the DRG explants, with axons actively penetrating and interweaving through the dense collagenous framework of the ANP. Concurrent staining for S100 (Schwann cell marker) demonstrated that Schwann cells not only adhered to the ANP surface but also migrated along the scaffold fibers, aligning closely with extending neurites to form a supportive glial network (Figure 3B–D). In comparison between the control group and the ANP group, the S100 fluorescent area in the ANP group was significantly higher than that in the control group, with the difference being statistically significant (Figure 3E). However, the difference in neurite outgrowth length between the two groups did not reach statistical significance (Figure 3F).

These observations indicate that ANPs preserve bioactive matrix cues and microstructural guidance channels conducive to axon–glia interactions. The close spatial association of Schwann cells with outgrowing neurites suggests that ANPs may promote myelination and stabilize regenerating axons in a manner similar to native nerve perineurium. Together, these results confirm that ANP scaffolds provide a permissive and structurally supportive microenvironment for neuronal adhesion, neurite extension and Schwann cell migration *in vitro*, underscoring their potential as a regenerative interface for peripheral nerve repair.

### ANPs remodel the configuration of the ECM at the site of injury to inhibit TN formation

The prepared ANPs membranes were trimmed into 3 mm × 3 mm sheets, sterilized by Co^60^ irradiation and applied in a sciatic nerve transection model in SD rats. A 2 mm segment of the sciatic nerve trunk was excised to create a gap, and microsurgical anastomosis was performed under a microscope to ensure optimal alignment before stump swelling occurred (Figure 3D and E). Operator feedback on the surgical procedure indicated that the ANP material did not experience any tearing during the operation. At 6 weeks postoperatively, the control group exhibited localized TN formation at the anastomosis site, characterized by swelling, scar hyperplasia and incomplete fascicular closure (Figure 3F). Clinically, even with meticulous microsuturing, the anastomosis often failed to achieve complete closure under persistent tissue tension (Figure 3G), inevitably leading to scar hyperplasia and subsequent neuroma formation (Figure 3H). In the ANP-treated group, the residual epineurium could be grasped with microforceps, allowing the flexible yet resilient ANP membrane to be affixed and sutured over the anastomosis without lifting (Figure 3I). At 6 weeks postoperatively, a translucent membrane without localized swelling was observed at the anastomosis site (Figure 3J and K). We hypothesize that the ANP membrane isolates the anastomosis from hematoma and surrounding muscle tissue, preventing pathological TN formation (Figure 3L and M).

**Figure 4:**
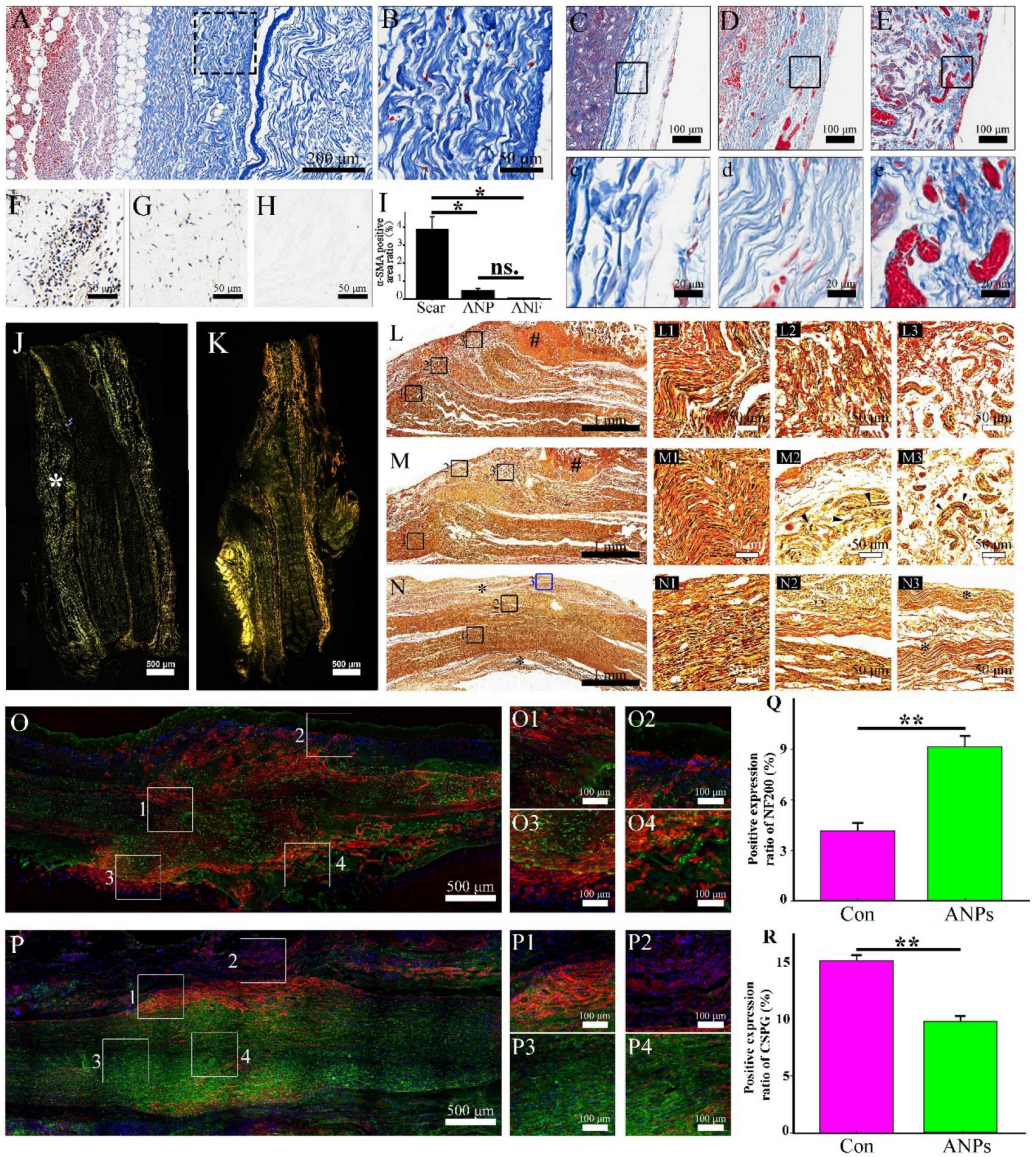
*In vivo* implantation experiments with ANAs and ANPs. ANPs optimize the regenerative microenvironment by remodeling the local extracellular matrix (ECM) at the injury site. (**A**) Masson’s trichrome staining of longitudinal sections of ANAs harvested 1 week after subcutaneous implantation in mice. (**B**) Higher magnification of (A), highlighting the fibrous epineurium layer of ANAs. (**C**) Longitudinal section of the crush-injury repair site stained with Masson’s trichrome. (c) Magnified view of the epineurium region in (C). (**D**) Longitudinal section of the anastomosis site in the ANP-treated group stained with Masson’s trichrome. (d) Magnified view of the ANP material in (D). (**E**) Longitudinal section of the anastomosis site in the control group stained with Masson’s trichrome. (e) Magnified view of the neuroma region in (E). (**F**–**H**) Immunohistochemical staining of α-SMA in tissues harvested 1 week after ANA implantation, showing scar formation in the extra-epineurium region (F), perineurium region (G) and nerve fascicle region (H). (**I**) Quantitative analysis of the α-SMA–positive area (*n* = 5 per group; data were presented as mean ± SD; one-way ANOVA followed by Bonferroni post hoc test; *P < 0.05, **P < 0.01, ***P < 0.001, ns: not significant). (**J**) Longitudinal section of the anastomosis site in the ANP-treated group stained with Sirius Red and imaged under polarized light microscopy (asterisk marks the anastomosis plane). (**K**) Longitudinal section of the anastomosis site in the control group under the same conditions as (J). (**L**) Silver staining of a superficial longitudinal section from the simple anastomosis repair group (# denotes muscle fiber invasion). (L1–L3) Higher magnifications of regions 1, 2 and 3 in (L). (**M**) Silver staining of a deep longitudinal section from the control group (# denotes muscle fiber invasion). (M1–M3) Higher magnifications of regions 1, 2 and 3 in (M; black arrows indicate nerve axons). (**N**) Silver staining of the repair site in the ANP-treated group. (N1–N3) Higher magnifications of regions 1, 2 and 3 in (N; asterisks indicate ANPs integrated with the autologous perineurium). (**O**) Distribution of NF200 and CSPG proteins at the anastomosis site in the control group. (O1–O4) Higher magnification views of regions 1–4 outlined in panel (O). (**P**) Distribution of NF200 and CSPG proteins at the anastomosis site in the ANP-treated group. (P1-P4) Higher magnification views of regions 1–4 outlined in panel (P). (**Q**) Quantitative analysis of the percentage of NF200-positive fluorescent area at the anastomosis site in control versus ANP-treated groups. (*n* = 5 per group; unpaired Student’s *t*-test; data were presented as mean ± SD; *P < 0.05, **P < 0.01, ***P < 0.001, ns: not significant). (**R**) Quantitative analysis of the percentage of CSPG-positive fluorescent area at the anastomosis site in control versus ANP-treated groups (*n* = 5 per group; unpaired Student’s *t*-test; data were presented as mean ± SD; *P < 0.05, **P < 0.01, ***P < 0.001, ns: not significant). Scale bars: A = 200 μm; B, F–H = 50 μm; C–E = 100 μm; c, d, e = 20 μm; J, K = 500 μm; L, M, N = 1 mm; L1–3, M1–3, N1–3 = 50 μm, O, P = 500 μm. O1-4, P1-4 = 100 μm.

Subcutaneous implantation of epineurium-stripped ANAs further confirmed the barrier function of ANPs. Masson staining showed that ANPs blocked invasion of external cells into nerve fascicles (Figure 4A and B). In the intact contralateral sciatic nerve of SD rats, the perineurium, a thin but dense connective tissue layer prevents nonneural tissue infiltration (Figure 4C). In the ANP group, the implanted ANPs integrated with host nerve tissue to form a barrier resembling the native perineurium (Figure 4D). In contrast, the control group exhibited disorganized collagen signals, loss of the normal morphology and erythrocyte stasis, indicative of aberrant vascular proliferation (Figure 4E). Immunohistochemical staining for α-SMA (a marker of TN) in subcutaneously implanted ANAs revealed significantly greater α-SMA signals in scar tissue outside the ANP layer than in the ANP layer and its interior (*P* < 0.05; Figure 4F–I), confirming that ANPs’ innate ECM barrier functions effectively inhibited scar invasion. Longitudinal sections of ANP-treated anastomoses stained with picrosirius red under polarized light revealed fusion of the ANP collagen framework with host nerve tissue at 6 weeks (Figure 4J), whereas controls displayed an irregular collagen distribution and hyperplastic abnormalities (Figure 4K). Silver staining of nerve fibers revealed disorganized, misdirected fiber sprouting in the controls (Figure 4L and M), with some regenerating fibers deviating from fascicular trajectories to form neuromas (black triangles, Figure 4L and M), indicating futile regeneration that is unlikely to reach target organs. In ANP-treated anastomoses, however, ANP integration restored a regenerative barrier (asterisk, Figure 4N), minimizing fiber dispersion and enabling directional regrowth along anatomical pathways (Figure 4N1–N3). Together, these results indicate that ANPs restore perineurial-like barrier function, suppress neuroma formation and facilitate directional axonal regrowth, thereby enhancing the likelihood of functional reinnervation. Furthermore, spatial redistribution of CSPGs, which are scar-inhibitory molecules, was observed. Longitudinal sections of control anastomoses revealed that CSPGs dispersed within regenerative channels (Figure 4O), with an inverse correlation between CSPG and NF200 (neurofilament) signals (Figure 4O1–4), confirming CSPG-mediated axonal growth inhibition. In contrast, ANP-treated anastomoses presented peripheral CSPG localization (Figure 4P) with robust intrafascicular NF200 expression (Figure 4P1–2), facilitating axonal regeneration (Figure 4P3–4). Quantitative analysis confirmed a significant reduction in the NF200 area in the control group (Figure 4Q) and a decrease in CSPG deposition in the ANP group (Figure 4R). Collectively, these results indicate that ANPs restore perineurial barrier integrity, limit inhibitory ECM deposition and establish a pro-regenerative microenvironment conducive to axonal growth.

### ANPs restore neural barrier stability and enhance the regenerative niche

**Figure 5:**
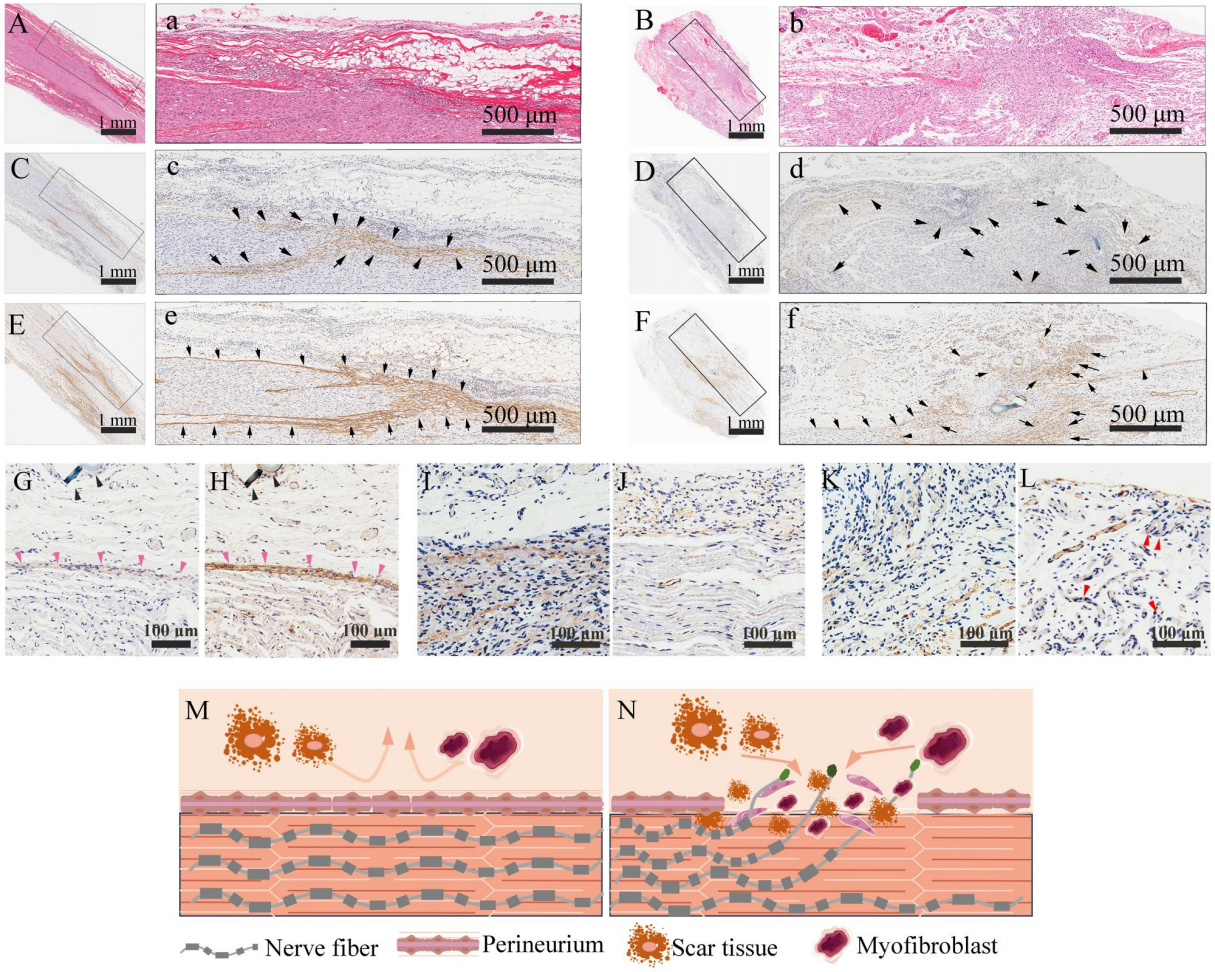
Histological evaluation of the neural anastomotic barrier. (**A**) Longitudinal section of the repair site in the ANP-treated group stained with Sirius Red. (a) Higher magnification of the boxed region in (A). (**B**) Longitudinal section of the repair site in the control group stained with Sirius Red. (b) Higher magnification of the boxed region in (B). (**C**) Longitudinal section of the repair site in the ANP-treated group after Claudin-1 immunohistochemistry (IHC). (c) Higher magnification of the boxed region in (C). (Arrows indicate positive signals). (**D**) Longitudinal section of the repair site in the control group after Claudin-1 IHC. (d) Higher magnification of the boxed region in (D). (Arrows indicate positive signals). (**E**) Longitudinal section of the repair site in the ANP-treated group after Glut-1 IHC. (e) Higher magnification of the boxed region in (E). (Arrows indicate positive signals). (**F**) Longitudinal section of the repair site in the control group after Glut-1 IHC. (f) Higher magnification of the boxed region in (F). (Arrows indicate positive signals). (**G**) Longitudinal section from the crush-injury group after Claudin-1 IHC (Arrows indicate positive signals). (**H**) Longitudinal section from the crush-injury group after Glut-1 IHC (Arrows indicate positive signals). (**I**) Longitudinal section from the ANP-treated group after S100 IHC. (**J**) Longitudinal section from the ANP-treated group after α-SMA IHC. (**K**) Longitudinal section from the control group after S100 IHC. (**L**) Longitudinal section from the control group after α-SMA IHC (Arrows indicate positive signals). (**M**) Schematic representation of perineurial barrier integrity in the ANP-treated and crush-injury groups: an intact perineurium prevents scar and myofibroblast invasion, maintaining a permissive microenvironment for axonal regeneration. (**N**) Schematic representation of the control group: barrier disruption allows scar tissue and myofibroblast infiltration, disturbing axonal regeneration and contributing to TN formation. Scale bars: A–F = 1 mm; a–f = 50 μm; G–L = 100 μm.

To assess the effect of ANP repair on neural barrier recovery, Sirius red staining was performed at the anastomosis site for both the ANP-treated and control groups. Bright-field imaging revealed minimal tissue extravasation in the ANP-treated group (Figure 5Aa), whereas the control group exhibited disorganized architecture and pronounced neuroma formation indicative of barrier disruption (Figure 5Bb). Immunohistochemistry for Claudin-1, a perineurial epithelial marker, showed a continuous, organized signal in ANP-treated nerves (Figure 5Cc) in contrast to the scattered and irregular signal distribution in controls (Figure 5Dd). Similarly, Glut-1 staining demonstrated polarized localization in the ANP group (Figure 5Ee) versus irregular distribution in controls, indicating impaired barrier restoration without ANP intervention. For comparison, crush injury sites exhibited intact Claudin-1 and Glut-1 patterns, consistent with preserved barrier function (Figure 5G and H). ANP-treated anastomoses also displayed fascicle-confined S100 expression (Figure 5I) and reduced α-SMA signals (Figure 5J), suggesting suppression of TN formation. By contrast, controls group presented diffuse S100 (Figure 5K) and increased α-SMA expression (Figure 5L), indicative of progressive neuroma pathology. These findings highlight the importance of rapid perineurial reconstruction in restoring barrier function, preventing scar tissue infiltration and preserving organized regenerative pathways (Figure 5M). Barrier compromise permits cellular invasion, which obstructs regenerative pathways or causes axonal extravasation (Figure 5N).

### ANPs enhance target axonal regeneration, muscle function and behavioral recovery

**Figure 6:**
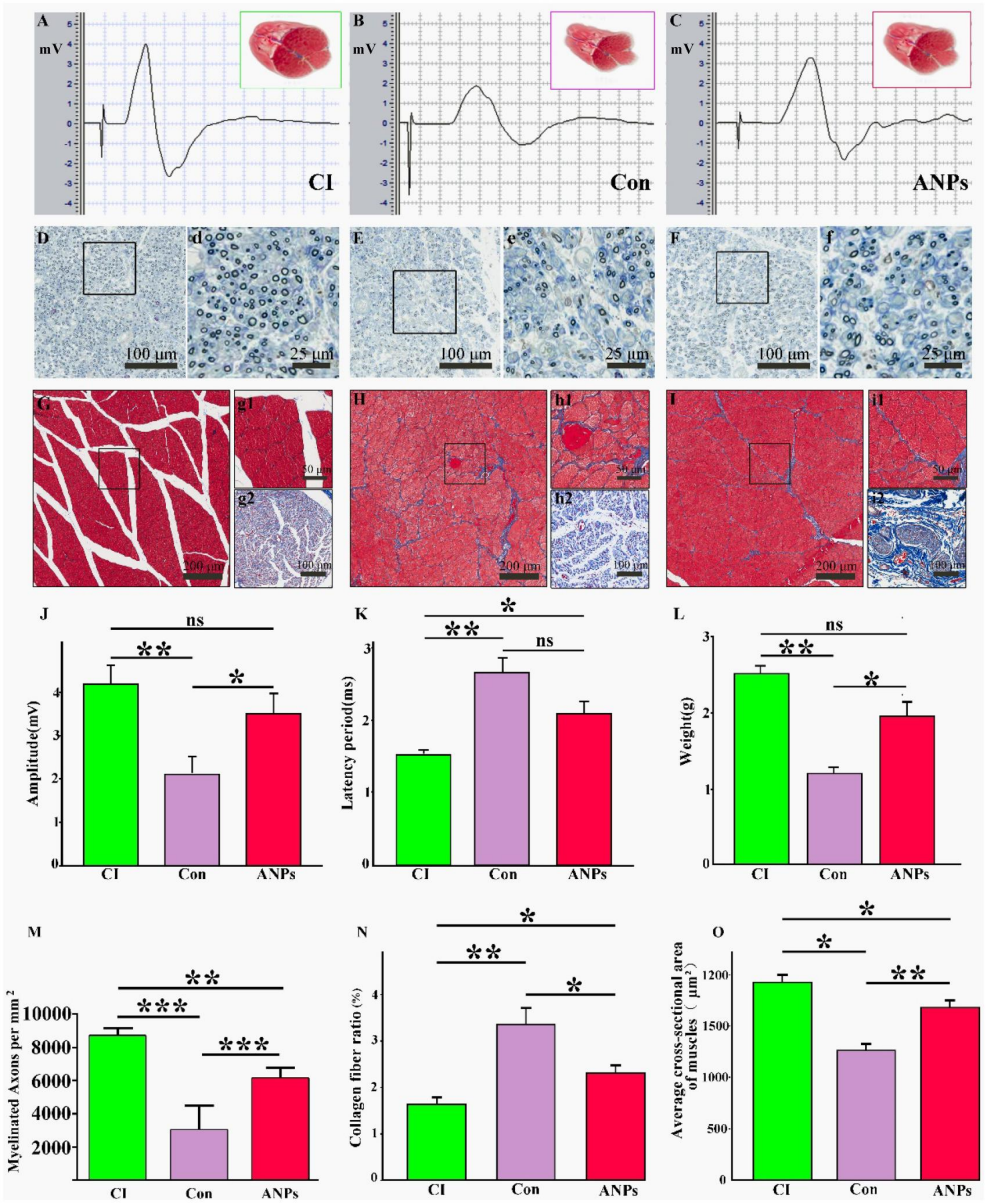
Histological and electrophysiological assessment of tibial nerve and gastrocnemius muscle recovery at 6 weeks post-surgery. Electrophysiological and histological analyses were performed to evaluate axonal regeneration, myelination and muscle reinnervation. (**A**–**C**) Representative compound muscle action potential (CMAP) waveforms recorded from the gastrocnemius in the crush injury group (A), control group (B) and ANP-treated group (C). (**D**–**F**) Myelin staining of tibial nerve cross-sections in the crush injury (D, d), control (E, e) and ANP (F, f) groups. Panels d, e, f shows magnified views of the boxed regions. (**G**–**I**) Masson’s trichrome staining of gastrocnemius cross-sections in the crush injury (G, g1, g2), control (H, h1, h2) and ANP (I, i1, i2) groups. Panels g1, h1, i1 show magnified views of muscle fibers and panels g2, h2, i2 highlight intramuscular nerve axons. (**J**–**O**) Quantitative analyses across groups: (J) CMAP amplitude, (K) CMAP latency, (L) gastrocnemius wet weight, (M) myelinated axon density in tibial nerves, (N) collagen area ratio in gastrocnemius and (O) muscle fiber area ratio. (*n* = 5 per group; one-way ANOVA followed by Bonferroni post hoc correction; data were presented as mean ± SD; *P < 0.05, **P < 0.01, ***P < 0.001, ns: not significant). Scale bars: D–F = 100 µm; d–f = 25 µm; G–I = 200 µm; g1, h1, i1 = 50 µm; g2, h2, i2 = 100 µm.

Because ANPs remodel the regenerative niche following peripheral nerve injury, we next evaluated whether these changes translated into improved axonal regeneration, muscle preservation and functional recovery. First, the conduction function of the tibial nerve and electrophysiological properties of the target muscle (gastrocnemius) were assessed in the crush injury group, control group and ANP group (Figure 6A–C). The gastrocnemius in the crush injury group exhibited the most robust morphology, with the highest amplitude and shortest latency of CMAPs (Figure 6A). In contrast, the control group presented significant gastrocnemius atrophy, accompanied by markedly reduced CMAP amplitude and nerve conduction velocity (NCV; Figure 6A). Compared with the control group, the ANP group presented minimal muscle atrophy, with significantly greater CMAP amplitudes and NCVs. Semithin sections of the tibial nerve adjacent to the muscle revealed abundant myelinated axons in both the crush injury and ANP groups, whereas myelinated axons were relatively rare in the control group (Figure 6D–F). To investigate how regenerated nerves ameliorate gastrocnemius atrophy, Masson’s trichrome staining was performed to characterize the morphology of the gastrocnemius and intramuscular regenerated nerves (Figure 6G–I). Nerve bundles containing numerous myelinated axons were detected in the muscles of the crush injury and ANP groups, whereas the control group presented scarce myelinated axons within nerve bundles (Figure 6G2–I2). The crush injury group retained the largest cross-sectional area of muscle fibers with minimal collagen deposition. Moreover, the ANP group presented significantly larger muscle fiber cross-sectional areas than did the control group. Statistical analysis revealed that the CMAP amplitude in the gastrocnemius muscle in the crush injury group was significantly greater than that in the control group but comparable to that in the ANP group (Figure 6J). The CMAP latency in the crush injury group was significantly shorter than that in the control group, whereas the latency in the ANP group was not significantly shorter than that in the control group (Figure 6K). Both the crush injury and ANP groups presented significantly greater gastrocnemius wet weights than did the control group, with no significant difference between the former two groups (Figure 6L). Although the density of myelinated axons in the tibial nerve in the ANP group was lower than that in the crush injury group was, it remained substantially greater than that in the control group (Figure 6M). The percentage of collagen area in the gastrocnemius muscle in the crush injury group was significantly lower than that in the control and ANP groups, whereas the ANP group presented a statistically significant reduction compared with the control group (Figure 6N). Conversely, the muscle area percentage in the crush injury group was significantly greater than that in the control and ANP groups, although the ANP group still outperformed the control group (Figure 6O). These results indicate that ANP implantation significantly improved the quality of axonal regeneration in target nerves after PNS injury, thereby reversing muscle atrophy and dysfunction. The narrowed gap between the ANP and crush injury groups suggests the potential for further functional recovery.

**Figure 7:**
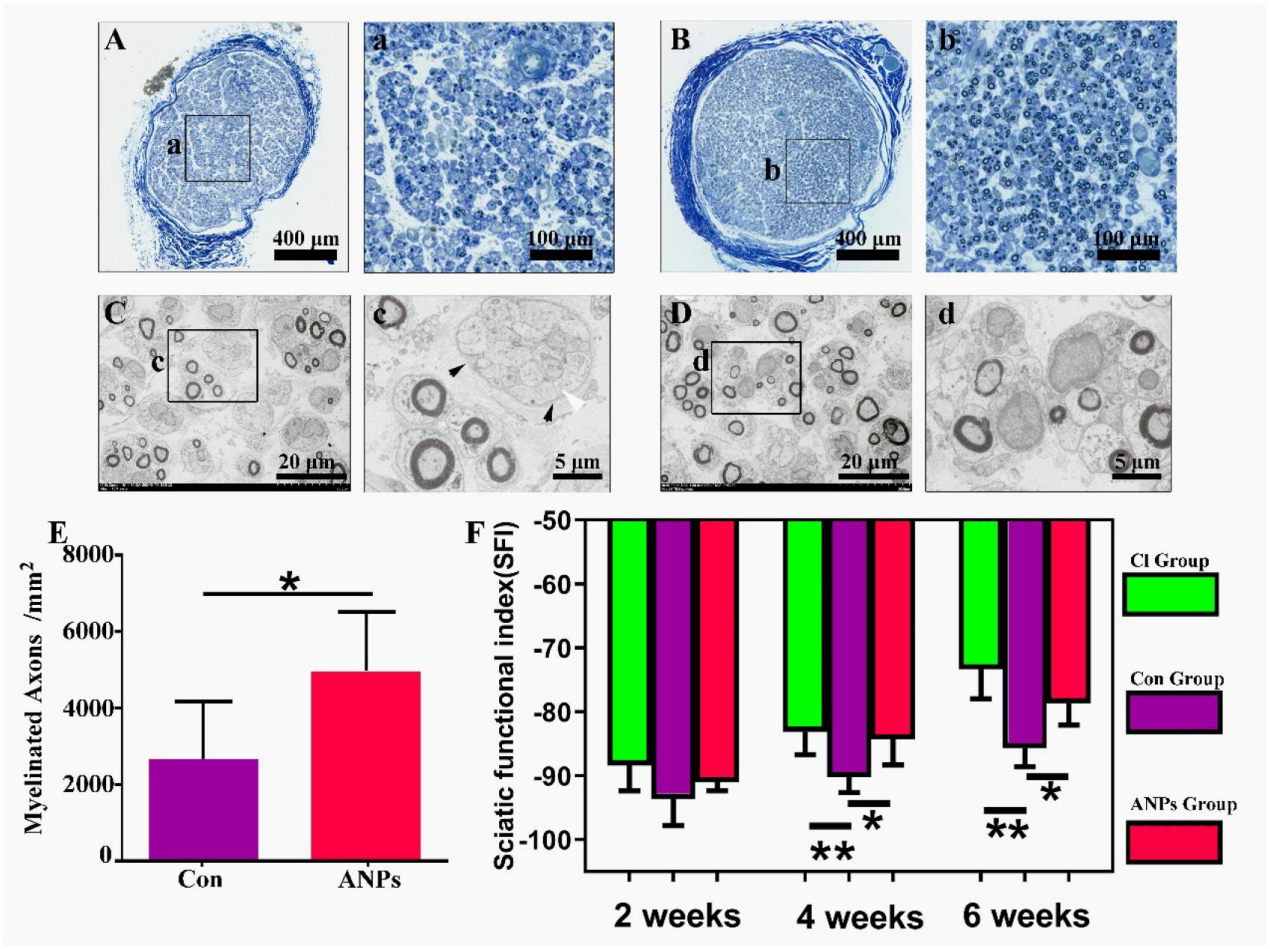
Distal myelinated axons in the common peroneal nerve and behavioral gait assessment. (**A**) Myelin staining of common peroneal nerve sections from the control group. (a) Magnified view of (A). (**B**) Myelin staining of common peroneal nerve sections from the ANP group. (b) Magnified view of (B). (**C**) SEM images of common peroneal nerve sections from the control group. (c) Magnified view of (C); black triangles indicate unmyelinated regenerated nerve bundles. (**D**) SEM images of common peroneal nerve sections from the ANP group. (d) Magnified view of (D). (**E**) Statistical analysis of myelinated axons (*n* = 5 for each group; unpaired Student’s *t* test; data were presented as mean ± SD; *P < 0.05, **P < 0.01, ***P < 0.001, ns: not significant). (**F**) Statistical comparison of sciatic functional index (SFI) values at 2-, 4- and 6-weeks post-surgery (*n* = 5 for each group; one-way analysis of variance (ANOVA) followed by Bonferroni *post hoc* correction; data were presented as mean ± SD; *P < 0.05, **P < 0.01, ***P < 0.001, ns: not significant). Scale bars: A, B = 400 µm; a, b = 100 µm; C, D = 20 µm; c, d = 5 µm.

Additionally, semithin sections of the common peroneal nerve adjacent to the tibialis anterior muscle were stained for myelin. The control group presented relatively few myelinated nerves (Figure 7Aa), whereas the ANP group presented a greater density of myelinated nerves (Figure 7Bb). SEM revealed that some regenerated basilar membrane tubes in the control group lacked regenerated nerves (Figure 7Cc), a phenomenon that was absent in the ANP group (Figure 7Dd). The difference in the density of myelinated axons between the two groups was statistically significant (Figure 7E). These findings suggest that ineffective regeneration in the control group prevented axons from reinnervating target organs. Finally, the sciatic functional index (SFI), which generates values ranging from 0 (normal function) to −100 (complete dysfunction), was used to assess motor recovery. SFI values were recorded biweekly from postoperative Week 2 to Week 6. All groups showed partial functional recovery over time, reflected by gradually increasing SFI values. However, starting in Week 4, the crush injury and ANP groups presented significantly greater SFI values than the control group, with further improvements thereafter. By Week 6, the ANP group achieved SFI values comparable to those of the crush injury group and significantly greater than those of the control group (Figure 7F), indicating substantial behavioral recovery. Collectively, these results demonstrate that ANPs substantially enhance the quality of axonal regeneration, reduce target muscle atrophy and improve both electrophysiological and behavioral recovery. The functional gap between ANP-treated and crush-injury groups narrowed over time, suggesting that ANPs not only mitigate the consequences of nerve transection but may also support near-normal reinnervation potential.

### ANPs modulate key molecular pathways to optimize axonal regeneration

**Figure 8:**
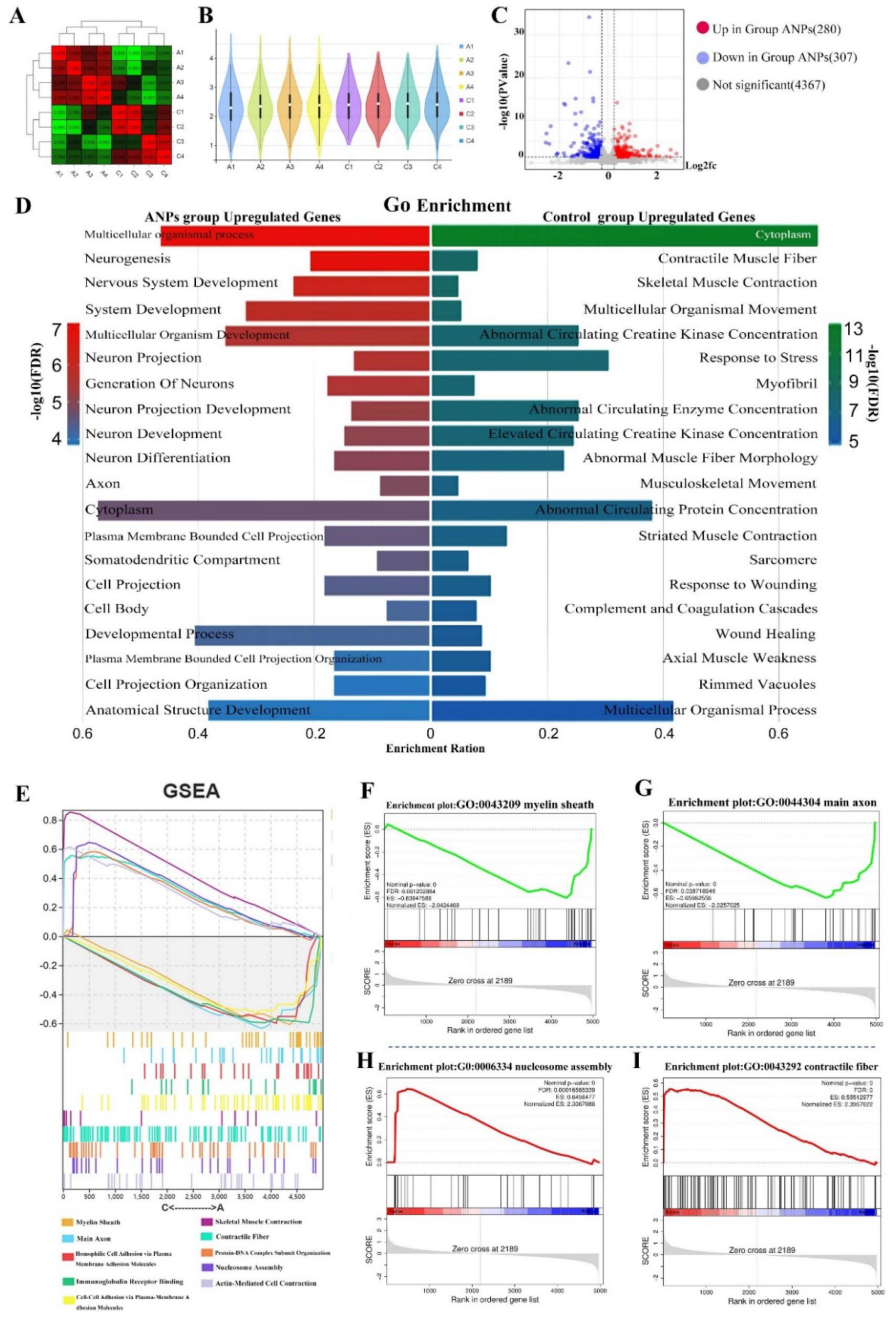
Proteomic analysis of regenerated sciatic nerve tissues distal to the anastomosis site at 6 weeks post-surgery. (**A**) Heatmap showing correlations between regenerated sciatic nerve tissues from the control and ANP-treated groups, illustrating distinct protein expression patterns. (**B**) Violin plot comparing the distribution of protein expression levels in regenerated sciatic nerve tissues between the control and ANP groups. (**C**) Volcano plot depicting differentially expressed proteins (DEPs) between the control and ANP groups. In the ANP group, 280 proteins were significantly upregulated and 307 proteins were significantly downregulated (*P*-value < 0.05, |log2(FC)| > 0.25). (**D**) Bar graphs presenting the top 20 Gene Ontology (GO) functional categories of the enhanced DEPs in the ANP group (the left panel) and control group (the right panel), respectively. (**E**) GSEA results for DEPs in regenerated sciatic nerve tissues between the control and ANP groups. (**F**, **G**) GSEA revealed enhanced functional items, including myelin sheath and main axon in the ANP group compared to the control group. (**H**, **I**) GSEA demonstrated enhanced functional items, including nucleosome assembly and contractile fiber in the control group compared with the ANP group.

To delineate the molecular mechanisms underlying the superior regenerative outcomes of ANP implantation, we performed a proteomic analysis of regenerated sciatic nerve segments distal to the anastomosis site at 6 weeks postoperatively. Correlation heatmapping revealed clearly segregated protein expression profiles between the ANP and control groups (Figure 8A), indicating distinct regulatory programs governing regeneration. Violin plots further demonstrated broader and more specific protein expression patterns in the ANP groups (Figure 8B). Volcano plot analysis revealed 280 significantly upregulated proteins and 307 significantly downregulated proteins in the ANP group compared with the control group (Figure 8C). These differentially expressed proteins (DEPs) are likely involved in critical regulatory processes of ANP-mediated nerve regeneration. GO functional analysis (Figure 8D) revealed that the upregulated proteins in the ANP group were enriched primarily in biological processes related to neural regeneration and development, axon guidance and cellular growth. In contrast, the upregulated proteins in the control group were predominantly associated with myofibril contraction, abnormal myofiber morphogenesis, multicellular organismal movement and musculoskeletal motion processes that are less relevant to peripheral nerve regeneration. Notably, the control group showed marked enhancements in the ‘response to stress’ and ‘response to wounding’ functional categories, suggesting heightened sensitivity to mechanical compression from surrounding muscles/scar tissues and chemical stimulation from inflammatory factors at the anastomosis site in the absence of ANP protection. Gene set enrichment analysis (GSEA) revealed significant enhancement of myelin sheath formation and the main axon regeneration pathways in the ANP group (Figure 8E–G), indicating superior myelination and axonal regrowth capacity. Conversely, the control group exhibited activation of predominantly nucleosome assembly and contractile fiber-related pathways (Figure 8H and I), potentially associated with impaired nerve regeneration and scar tissue formation. Collectively, our proteomic findings demonstrate that ANPs promote nerve regeneration and functional recovery through the coordinated regulation of myelination and axonal regeneration pathways. The upregulated proteins in the ANP group likely participate in myelin repair and axonal regrowth, whereas the downregulated proteins may be involved in inflammatory responses and fibrotic scarring. Collectively, these proteomic findings demonstrate that ANPs promote peripheral nerve repair by simultaneously upregulating proteins involved in myelination and axonal regrowth while downregulating stress- and fibrosis-related mediators. This coordinated molecular reprogramming provides mechanistic evidence that ANP scaffolds act as multitarget modulators of the regenerative niche, driving functional recovery through enhanced neural repair rather than compensatory tissue responses.

## Discussion

Prior to initiating the development of human ANAs, our team conducted nonhuman primate experiments to investigate the feasibility of using ANAs to repair TPNIs [17]. During that era, when tissue engineering was still in its infancy, we pioneered the exploration of novel approaches for repairing TPNI defects. Since then, ANA transplantation has become a mainstream technique for reconstructing long-segment TPNI defects in multiple countries, providing an alternative to autologous nerve grafts and demonstrating stable and definitive therapeutic outcomes [8, 18]. Decellularization eliminates the immunogenic risks of ANAs while preserving the ECM architecture of PNS tissue. The resulting biomechanical support and biochemical signaling effectively promote axonal regeneration, cell migration and tissue remodeling in humans [1, 19]. We propose that the successful repair of TPNI defects by ANAs stems from their biomimetic properties, which achieve the microanatomical matching required for PNS regeneration. The regenerative channels formed by nerve fascicles within ANAs and structural support from the perineurium exhibit precise matching with damaged host nerves across the macroscopic anatomy, microstructure and ECM composition. The unique PNS-specific ECM framework of ANAs undergoes tissue integration and inductive remodeling processes, ultimately assimilating with autologous nerves and initiating sustained TPNI regeneration. The significance of ANAs extends beyond clinical repair—they represent a paradigm shift because they have advanced theoretical knowledge related to neural tissue engineering and regenerative medicine. For example, inspired by ANAs, numerous ANA matrix-derived materials have been developed, including injectable decellularized nerve hydrogels that retain the axonal regeneration-promoting and myelination capacities of ANAs. These issues have become research hotspots in the context of both central and PNS injury [20–23]. Another example is nerve protective membrane materials derived from porcine ANAs, which have been successfully translated for clinical application in TPNI patients without substantial tissue defects [24]. Given the diverse clinical presentations of TPNI encountered by surgeons, refinement of established technologies remains imperative for developing novel therapeutic approaches. We maintain that ANA-derived materials hold promise for further improving motor and sensory function recovery in TPNI patients.

The barrier structure of ANAs has recently attracted significant research attention. In this study, we propose a novel concept of neural barrier repair using ANPs. Derived from the perineurial barrier structure of ANAs, this barrier repair material can accelerate the restoration of physiological barrier structures in TPNI through tissue integration and induced remodeling, effectively improving repair outcomes in rat sciatic nerve transection models under low neural tension conditions. As derivatives of ANAs, ANPs meet the microanatomical matching requirements for PNS repair and can be clinically applied to TPNI cases with disrupted neural barriers, potentially reducing the failure rate of primary surgical repair. We systematically investigated the microstructure of ANPs, their ECM omics characteristics, and their capacity to restore physiological barrier integrity after TPNI. The microstructure and ECM omics profile of ANPs closely resemble those of the autologous perineurium but differ markedly from those of the endoneurial basement membrane ECM of nerve fascicles. Consequently, ANPs demonstrate repair capabilities beyond mere antiadhesion effects—when applied to TPNI sites, they rapidly regenerate and integrate to repair damaged neural barriers. This evidence confirms that ANPs preserve and replicate the microanatomical matching properties of ANAs for PNS repair. It has been consistently observed that perineurium rupture leads to endoneurium segmentation into regenerating fiber ‘microfascicles’ surrounded by perineurium-like cells, thereby initiating trauma repair programs [25]. These findings demonstrate that the perineurium serves as a critical structure for maintaining PNS physiological status and postinjury endoneural homeostasis. Recent studies have revealed that orderly perineurium reconstruction rapidly restores endoneural environmental homeostasis through guided matrix remodeling, prevents edema caused by vascular leakage and facilitates the transformation of fibrotic wounds into permissive bridging tissue supporting axonal regeneration [26]. In this study, we explicitly identified the perineurial barrier structure of the PNS as a therapeutic target of ANPs. Previous research has shown that the perineurium exhibits distinct microstructural characteristics, with its primary difference from the endoneurium being the absence of basement membrane tubes secreted by Schwann cells [16, 27]. Through meticulous dissection of ANAs to remove nerve fascicles (containing endoneurial basement membrane ECM) and epineurial tissue, we successfully prepared ANPs. Microstructurally, ANPs primarily consist of longitudinally aligned nanoscale ECM fibers, with their inner walls displaying relatively smooth interfacial characteristics. Although the decellularization process caused some structural damage, the native perineurial architecture remained largely preserved.

The spatial distribution of various cell types in the PNS exhibits clear anatomical specificity: Schwann cells are primarily localized within the endoneurium, where they form the basal lamina structure [28], whereas perineurial epithelial cells are specifically distributed in the perineurium [29]. On the basis of these anatomical characteristics, we hypothesize that the ECM composition in PNS tissues is anatomically compartmentalized. Proteomic analysis revealed that, compared with the endoneurial basal lamina, ANAs are relatively deficient in pro-neurite outgrowth factors, particularly as evidenced by the significantly reduced content and diversity of basal lamina components such as laminin. This finding confirms substantial differences in microstructure and compositional organization among different tissue compartments of ANAs, which correspond to distinct biological functions. These results indicate that ANPs function primarily in regulating the mechanical microenvironment and forming the blood-nerve barrier. The ECM proteins in ANPs are predominantly involved in biological processes, including protein polymerization, platelet activation, endoplasmic reticulum function, fibrinogen complex formation, carbohydrate binding, the Golgi apparatus and mitochondrial activities. These functional characteristics align with the roles of these proteins in protein synthesis, nutritional supply and vascular enhancement and are consistent with previously reported functions of isolating external stimuli and preventing peripheral neuropathies [30–32]. In contrast, the nerve fascicle region provides individualized compartmental spaces for each axon, with ECM components more specialized in promoting axonal growth and extension. By using proteomic sequencing technology, we successfully detected all protein peptides in both ANFs and ANPs. The results demonstrated that peptides associated with nerve fibers and the basal lamina were predominantly present in ANF tissues, suggesting that ANFs constitute the functional core region of the PNS, whereas ANPs primarily serve as barrier structures. Overall, the biological functions of the protein peptides strongly correlated with the ECM protein profiles. Overall, ANPs exhibit limited efficacy in directly promoting nerve regeneration, both in terms of microstructural features and ECM composition. However, ANPs retain fundamental capabilities in the regulation of neural microenvironments because of their abundant matrix components, including polysaccharides and glycoproteins. This study represents the first observation of anatomically defined ECM heterogeneity in the PNS, a phenomenon previously documented in decellularized lung, bone, skin, intervertebral disc, brain and meniscus studies [33–38]. This discovery provides novel perspectives for understanding the structural and functional characteristics of the PNS.

Our research team previously successfully prepared rat-derived ANPs [11]. We found that ANPs possess moderate mechanical strength and toughness, indicating that the technical procedure of harvesting ANPs after loosening the epineurium is both feasible and operationally straightforward. Following immersion in water, ANPs become pliable and can conform tightly to nerve anastomosis sites, requiring only two microsutures for secure fixation. Studies have reported Young’s modulus values of 3.0 ± 0.3 MPa for the perineurium and 0.4 ± 0.1 MPa for the epineurium [39], indicating that ANPs exhibit superior stiffness that enhances resistance to external mechanical stress. In this study, we compared the mechanical properties of ANPs and intact ANAs. Our findings reveal that although ANPs exhibit lower tensile strength than ANAs, their elongation at break is comparable, and their suture retention strength shows no significant difference. ANPs, thus, present an optimal mechanical balance: sufficient strength to serve as a structural scaffold for the neural barrier, coupled with high extensibility that provides dynamic biomechanical protection. When surgeons wrap a nerve anastomosis with ANPs, they can be confident in its mechanical reliability—concerns regarding suture failure or early graft disruption due to material weakness are alleviated. This reliability significantly enhances the translational feasibility of this technique.

In clinical nerve repair surgeries, tension at the anastomosis site frequently occurs due to necessary debridement of nerve endings, which may precipitate perineurial window effects, leading to TN formation. This complication can compromise repair outcomes and potentially necessitate revision surgery. The ANPs we developed demonstrate a remarkable capacity to prevent scar tissue invasion and suppress abnormal ECM remodeling at anastomotic sites. Following implantation, ANPs are assimilated into the autologous perineurium, rapidly restoring neural homeostasis and significantly improving nerve regeneration outcomes. These findings are corroborated at the molecular level through proteomic analysis of *in vivo* experiments. Compared to the Con group, the ANP group showed upregulation of gene expression in multiple functional categories closely related to peripheral nerve regeneration, including neurogenesis, nervous system development, neuron projection, neuron differentiation and axonogenesis. In contrast, the Con group exhibited elevated expression in functional categories related to myofibril contraction, musculoskeletal movement, stress response and wound healing. Histological staining results indicated that in the absence of ANPs, delayed restoration of nerve barrier function at the suture site increased susceptibility to mechanical compression from surrounding muscle/scar tissue and inflammatory factors during regeneration, exacerbating scar proliferation and resulting in suboptimal neural regenerative activity. These observations are consistent with the histological and functional outcomes in animal models, reflecting incomplete neural regeneration during scar formation at the anastomosis in controls. It is noteworthy that proteomic and bioinformatic analyses provide deeper molecular mechanistic explanations for the phenotypic differences described above. Our proteomic analysis revealed that ANPs are enriched in ECM proteins associated with ‘platelet activation’, ‘coagulation’, ‘wound healing’ and ‘cell adhesion’ (e.g. VWF, VTN, fibrinogen complex components). These components are not merely structural scaffolds but also active signaling reservoirs. For instance, VWF and VTN are known integrin ligands (e.g. αvβ3, αvβ5) [40]. Following nerve injury, the enrichment of these proteins in ANPs may guide Schwann cell migration, proliferation and morphological remodeling via the integrin-FAK-Rac/Rho GTPase signaling axis, promoting their transition from a quiescent myelinating phenotype to a pro-regenerative repair phenotype. Hallmarks of this phenotypic shift include the upregulation of neurotrophic factors (e.g. NGF, BDNF) and cell adhesion molecules (e.g. L1, N-cadherin). Our proteomic data showed an upward trend in molecules related to ‘neurotrophic factor signaling pathway’ and ‘cell adhesion’ in the ANP group, providing indirect support for this mechanism. GSEA analysis demonstrated that ANPs significantly promote myelin sheath synthesis and axonal growth (main axon), which aligns with GO functional analysis results indicating enhanced activity related to neurofilament synthesis, myelination and nervous system development in the ANP group. In contrast, the control group predominantly expressed proteins associated with contractile fiber function, DNA-protein complex formation and nucleosome assembly, indicating persistent scar proliferation and incomplete neural regeneration. These findings suggest that ANPs, through their unique ECM components (such as enriched integrin ligands like VWF and VTN), may activate integrin signaling pathways (e.g. αvβ3, αvβ5) on Schwann cells and neurons, thereby promoting cell migration, proliferation and axonal growth via downstream pathways like FAK-PI3K/Akt, while simultaneously suppressing abnormal ECM deposition and stress response pathways that lead to scarring. Future studies will employ *in vitro* Schwann cell-ANP co-culture models to directly assess integrin subunit activation, downstream FAK phosphorylation and expression changes in repair phenotype markers to validate this hypothesis.

As integral components of ANAs, ANPs exhibit optimal biocompatibility with the regenerative demands of autologous neural tissue. Unlike ECM-based membrane materials that undergo hydrolytic processing, ANPs circumvent concerns related to *in vivo* degradation, substantially reducing application complexity. Importantly, omics analyses predicted—and subsequent *in vivo* experiments confirmed—the capacity of ANPs to facilitate neural barrier restoration. Claudin-1 and Glut-1 are known as critical molecular constituents of the blood-nerve barrier (BNB): Claudin-1 maintains structural integrity via tight junction formation, while Glut-1 sustains neuronal energy supply through glucose transport regulation [41, 42]. Both proteins play profound roles in the PNS, where aberrant expression or distribution may contribute to neuropathogenesis [43]. Under ANP intervention, coordinated immunohistochemical patterns of Claudin-1 and Glut-1 were observed at neural anastomoses, indicating progressive normalization of the barrier architecture. We posit that ideal adjunctive biomaterials for PNS surgical repair should possess this capability, given that TPNI universally involves barrier disruption—where rapid stabilization is pivotal for mitigating neuropathic pain and enhancing sensorimotor recovery.

A crush injury control group was included in the experimental design. Due to preserved perineurial continuity, this TPNI model typically permits faster functional recovery in animal studies [44]. Remarkably, the histological, functional and behavioral outcomes following complete transection with ANP intervention were similar to those of the crush injury controls—a finding consistent with recent reports on other protective nerve wraps. ANPs not only accelerated organized barrier regeneration at anastomotic sites but also markedly suppressed the expression of chondroitin sulfate proteoglycans (CSPGs) [45], key inhibitory molecules for axonal regrowth. More importantly, CSPGs exhibited spatial redistribution, reflecting niche remodeling during regeneration. Our prior work demonstrated that spatially patterned CSPGs facilitate axonal guidance [46]. The observed confinement of CSPGs to superficial injury zones rather than regenerative pathways suggests enhanced axonal penetration of target organs. Although multiple CSPG-targeted regenerative strategies exist [47], our results indicate that ANPs reconstitute neural homeostasis by modulating the local CSPG distribution pattern. Certain matricellular proteins enriched in ANPs according to our proteomics (e.g. ITIH family proteins) have been reported to stabilize ECM and regulate protease activity. They may influence CSPG degradation and remodeling by modulating the activity of matrix metalloproteinases (MMPs) or aggrecanases (ADAMTS), thereby ‘confining’ CSPGs to specific regions. The activation of pathways related to ‘protease inhibitor activity’ in the ANP group in KEGG pathway enrichment analysis supports this possibility. Additionally, pro-adhesive ECM proteins provided by ANPs (such as specific fragments of laminin and fibronectin) may activate integrins (e.g. α6β1, α7β1) on axonal growth cones, initiating pro-growth PI3K-Akt and FAK-MAPK signaling pathways, thereby counteracting CSPG-mediated inhibitory signals. Proteomic data showed an upward trend in proteins related to the ‘PI3K-Akt signaling pathway’ and ‘regulation of actin cytoskeleton’ in regenerated nerves of the ANP group, which may represent the molecular basis for ANP-promoted axonal traversal through CSPG-enriched zones. Therefore, ANPs not only function as a passive barrier through their structural properties but also actively ‘isolate’ inhibitory signals like CSPGs from regenerative channels via ECM remodeling, while their enriched pro-adhesive ECM components may provide alternative, pro-growth integrin signals for growth cones, thereby synergistically optimizing the regenerative microenvironment. Next, we aim to functionally validate the necessity of ANPs regulating these specific signaling pathways (e.g. integrin-FAK, PI3K-Akt) to more precisely elucidate their molecular mechanisms. In summary, through ECM remodeling, ANPs concurrently restore BNB functionality and ensure sustained neural protection, offering novel therapeutic insights for peripheral nerve repair.

Previous studies have engineered protective interfacial barriers using materials such as the intestinal mucosa, amniotic membrane, epineurium and collagen to isolate nerves from surrounding tissues [48–51]. However, no reports exist regarding ANPs derived from native PNS barrier structures as neural protective materials. Although the ANA perineurium shows promise in mitigating TN formation, its role in regulating regeneration remains unclear. Our work innovatively develops animal-derived ANPs and elucidates their niche-modulating functions. Several limitations warrant acknowledgment. First, comparative analyses with other protective wraps are lacking. Second, the feasibility of human-derived ANP preparation remains unexplored. Third, the therapeutic efficacy of ANPs must be validated in large animal models. The successful clinical translation of ANPs will necessitate addressing these challenges in future studies.

## Conclusion

Building on the established ANA platform, we developed an ANP scaffold as a novel strategy for repairing TPNI. Using the same preparation techniques as for ANAs, ANPs were, for the first time, applied to reinforce low-tension anastomoses after sciatic nerve transection. This study demonstrates that ANPs optimize the ECM microenvironment, restore neural barrier integrity and enhance axonal regeneration, ultimately supporting functional recovery. The key findings are as follows: ECM remodeling and barrier repair—ANP implantation promoted perineurial barrier reconstruction through ECM remodeling, reduced the deposition of inhibitory CSPGs and mitigated scarring at the repair site. Promotion of nerve regeneration—ANPs increased the density of myelinated axons, preserved target muscle morphology and improved electrophysiological and behavioral recovery compared with controls. Mechanistic insights from multiomics—proteomic analyses revealed that ANPs establish a protective regenerative interface by enhancing neurofilament synthesis, myelination and structural stability, while downregulating pathways linked to inflammation and fibrosis. This molecular reprogramming transforms the anastomosis site from a fragile, injury-prone niche into a permissive interface capable of sustaining long-term axonal regrowth.

Collectively, these findings provide experimental evidence supporting ANPs as a multitarget, biomaterial-based therapy for peripheral nerve repair. Given their off-the-shelf manufacturability, compatibility with existing microsurgical techniques, and ability to modulate multiple regenerative pathways simultaneously, ANPs represent a promising translational biomaterial for clinical nerve reconstruction and functional restoration in complex peripheral nerve injuries.

## Data Availability

The mass spectrometry proteomics data have been deposited to the ProteomeXchange Consortium (https://proteomecentral.proteomexchange.org) via the iProX partner repository with the dataset identifier PXD071986. Data will be made available on request.
